# Congenital blindness reduces myelination in human visual cortex

**DOI:** 10.1126/sciadv.aec2348

**Published:** 2026-07-10

**Authors:** Anna-Lena Stroh, Luke J. Edwards, Daniel Haenelt, Fakhereh Movahedian Attar, Kerrin J. Pine, Robert Trampel, Marcin Szwed, Nikolaus Weiskopf

**Affiliations:** ^1^ Institute of Psychology, Jagiellonian University, ul. Ingardena 6, 30-060 Kraków, Poland.; ^2^ Department of Neurophysics, Max Planck Institute for Human Cognitive and Brain Sciences, Stephanstraße 1a, 04103 Leipzig, Germany.; ^3^ Cognitive Neuroscience, Faculty of Psychology and Neuroscience, Maastricht University, Oxfordlaan 55, 6229 EV Maastricht, The Netherlands.; ^4^ Institute for Anatomy I, Medical Faculty & University Hospital Düsseldorf, Heinrich-Heine-University Düsseldorf, 40225 Düsseldorf, Germany.; ^5^ Institute of Neuroscience and Medicine (INM-1), Research Centre Jülich, 52428, Jülich, Germany.; ^6^ Wellcome Centre for Human Neuroimaging, UCL Queen Square Institute of Neurology, University College London, 12 Queen Square, London WC1N 3AR, UK.; ^7^ Felix Bloch Institute for Solid State Physics, Faculty of Physics and Earth System Sciences, Leipzig University, Linnéstraße 5, 04103 Leipzig, Germany.

## Abstract

Sensory experience is critical for cortical maturation, but the cellular consequences of its absence remain poorly understood in humans. Using in vivo sub-millimeter 3 T and 7 T MRI and ultra-high-gradient diffusion MRI, we investigated the effects of congenital blindness on the human early visual cortex. Blind individuals showed reduced *R*_2_^*^ and *MT_sat_*—markers of iron and myelin—along with increased diffusivity, orientation dispersion, and reduced neurite density in the gray and superficial white matter. Cortical thickness was increased in blind individuals and associated with lower myelin and iron, questioning the long-standing assumption that increased thickness primarily reflects disrupted pruning. Our results provide no direct evidence for disrupted pruning. However, they suggest reduced myelination and oligodendrogenesis as key effects of congenital blindness and highlight the critical role of sensory input in shaping and stabilizing cortical circuits.

## Teaser

Altered myelin and microstructure in congenital blindness illuminate how sensory experience guides human brain development.

## INTRODUCTION

In humans, the first years of life are characterized by rapid brain development. In the first year alone, cortical surface area expands by 76% and cortical thickness increases by 31% (*1*). These structural changes are the result of precisely regulated molecular and cellular mechanisms (*2*), such as neurogenesis and neuronal migration (*3*), astrogliogenesis (*4*, *5*) and oligodendrogenesis (*6*), synaptogenesis (*7*, *8*), pruning (*7*–*9*), and myelination (*10*–*12*). These coordinated processes shape all mammalian brains, but their prolonged course in humans, particularly myelination (*11*), may underlie our exceptional cognitive capacities.

A key unresolved question is the extent to which sensory experience during early development shapes these processes. The study of congenitally blind individuals can provide valuable insights into this question. Structural MRI studies have consistently reported increased cortical thickness in early visual areas (V1–V3) of congenitally blind individuals (*13*–*16*). In sighted individuals, imaging studies of infants and older children have shown that cortical thickness increases during the first two years of life, after which it thins to adult levels (*1*). It is widely assumed that developmental pruning—a process in which excess neural connections are eliminated—plays a central role in cortical thinning during development. Thus, the prevailing view is that a lack of visual experience may interfere with pruning processes, resulting in increased cortical thickness in blind individuals (*13*, *14*). However, emerging evidence challenges the notion of cortical thinning during development. Instead, it suggests that, depending on the cortical area in question, apparent cortical thinning may reflect increased intracortical and subcortical white matter myelination, which alters MRI contrast and makes the cortex appear thinner (*10*). Thus, previous observations of apparent thicker visual cortex in blind individuals may reflect reduced myelination. These mechanisms are not mutually exclusive, and a combination of reduced pruning and/or reduced myelination may result in apparently thicker cortex in congenitally blind individuals.

Conventional MRI lacks the necessary spatial resolution and tissue specificity to disentangle these microstructural mechanisms (*17*). However, advanced quantitative (qMRI) (*17*) and diffusion (dMRI) (*18*–*20*) MRI now make this feasible as they allow estimation of tissue properties at unprecedented spatial resolutions in vivo (*17*, *21*). Diffusion MRI is sensitive to the microstructural properties of brain tissue (*17*, *22*), and models such as NODDI (neurite orientation dispersion and density imaging) can estimate neurite density and orientation dispersion, providing insight into the organization of axons and dendrites within grey and white matter (*23*). Using multi-parameter mapping (MPM), the longitudinal relaxation rate (*R_1_*), effective transverse relaxation rate (R_2_^*^), magnetization transfer saturation (*MT_sat_*), and proton density (*PD*) can be quantified as sensitive markers of iron and myelin (*17*, *24*–*26*). Complementary dMRI metrics, including mean diffusivity (*MD*), radial diffusivity (*RD*) and axial diffusivity (*AD*) (*27*, *28*), as well as neurite density (*NDI*) and orientation dispersion (*ODI*) (*23*), offer additional insights into myelination and fiber organization. Together, these multimodal imaging techniques provide a detailed and non-invasive view of brain microstructure.

**Fig. 1.**
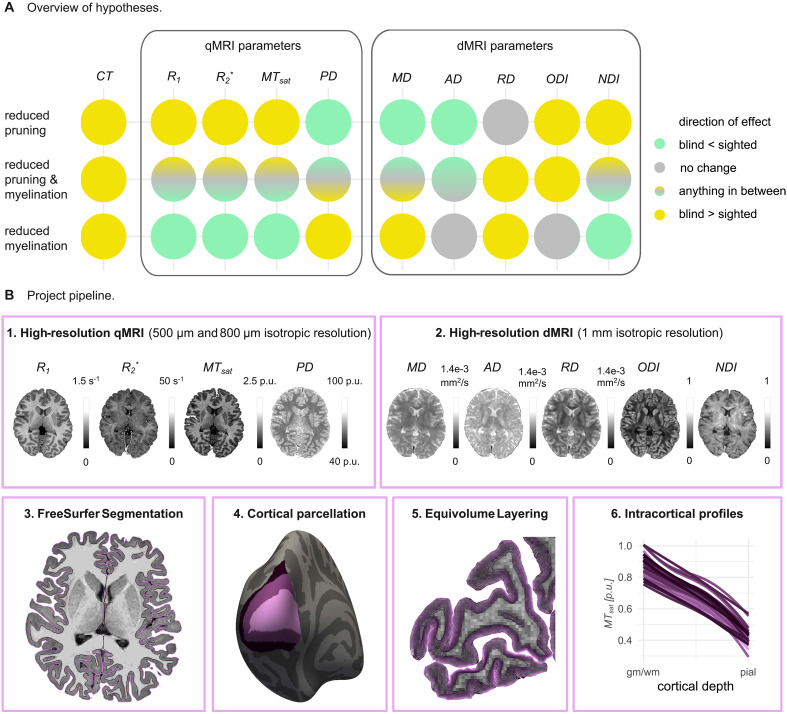
Linking altered neurodevelopment in congenital blindness to qMRI and dMRI metrics: Hypotheses and project pipeline. (**A**) Hypotheses. If reduced pruning predominates, we expect increased *R_1_*, R_2_^*^, *MT_sat_*, *ODI*, and *NDI* (yellow) in blind compared to sighted individuals, along with reduced *PD*, *MD*, and *AD* (mint). If reduced myelination predominates, we expect reduced *R_1_*, R_2_^*^, *MT_sat_*, and *NDI* in blind compared to sighted individuals; along with increased *PD*, *MD*, and *RD*. We cannot rule out concurrent effects of reduced pruning and myelination in congenital blindness, in which case we would expect increased *RD* and *ODI* in blind individuals. Moreover, this could result in either increased *R_1_*, R_2_^*^, *MT_sat_*, and *NDI*, accompanied by decreased *PD* and *MD*, or decreased *R_1_*, R_2_^*^, *MT_sat_*, and *NDI*, with increased *PD* and *MD*, depending on which of the two mechanisms predominates (mint-yellow gradient). (**B**) Methods overview. Ultra-high resolution quantitative parameter mapping was performed at 3 T and 7 T. Example 3 T quantitative parameter maps of a sighted participant are shown. Ultra-high-gradient-strength diffusion MRI was performed at 3 T. Example parameter maps of a sighted participant are shown. FreeSurfer was used for brain tissue segmentation. Regions of interest (ROIs; V1, V2, V3) were defined using the Benson atlas (*38*). We used equivolume-layering to sample qMRI and dMRI parameters from different cortical depths and to produce intracortical profiles of those parameters. *CT* = cortical thickness; Quantitative MRI parameters: *R_1_* = longitudinal relaxation rate, R_2_^*^ = effective transverse relaxation rate, *PD* = proton density, *MT_sat_* = magnetization transfer saturation. Diffusion MRI parameters: *RD* = radial diffusivity, *AD* = axial diffusivity, *MD* = mean diffusivity, *NDI* = neurite density index, *ODI* = orientation dispersion index. Hypotheses preregistered at: https://osf.io/8d2wr/.

Here, we investigated the alterations in tissue microstructure in the human early visual cortex and its underlying superficial white matter (SWM) in congenitally blind individuals. We hypothesized that congenital blindness may affect one or both of the following developmental processes: pruning and/or myelination. Reduced pruning is expected to be reflected in increased macromolecular content (*10*), reduced diffusivity measures (*10*), and increased complexity in the local fiber architecture and density, whereas reduced myelination is expected to be reflected in reduced myelin (*10*, *29*), increased diffusivity measures (*30*–*32*), and reduced complexity in the local fiber architecture (*33*) and density (*34*). Based on previous studies (*35*), we hypothesized that group differences—across all microstructural and morphological measures—would be most pronounced in V1, with decreasing effects from V1 through V3. The distinct predictions of each hypothesis across qMRI and dMRI parameters were preregistered at https://osf.io/8d2wr/, summarized in Fig. 1. The rationale for the preregistered hypotheses is further detailed in the Supplementary Text.

To test these hypotheses, we acquired qMRI and dMRI data with unprecedented image quality and spatial resolution by leveraging recent advances in imaging techniques and specialized acquisition protocols, including optical prospective motion correction (*25*, *36*, *37*), on cutting-edge 7 T (*24*) and high-performance gradient 3 T Connectom (*18*–*20*) MRI scanner platforms, in congenitally blind individuals and matched sighted controls. Using biophysical modeling, we examined microstructural properties of the cortex and superficial white matter in early visual areas V1–V3. We then evaluated how these measures relate to cortical thickness. We provide in vivo evidence that the absence of visual experience reduces myelination. The results question the prevailing view that pruning is the primary driver of structural variations in congenital blindness and argue for a broader perspective that includes myelination as a central factor.

## RESULTS

To investigate how visual experience influences cortical maturation of the early visual cortex in humans, we compared microstructural properties of V1, V2, and V3 in congenitally blind individuals (*n* = 24; female/male: 10/14, mean age: 35.01 years, age range: 19.2–47.2 years) and sighted controls matched for age and sex (*n* = 24; female/male: 10/14, mean age: 34.32 years, age range: 22.11–48.7 years). Effective group sizes for each analysis are provided in tables S1 and S2. High-resolution qMRI and dMRI data were acquired using a state-of-the-art 7 T MRI system and a high-performance gradient 3 T Connectom scanner. We defined regions of interest (ROIs) corresponding to V1, V2, and V3 using the Benson atlas (*38*), which predicts the locations and retinotopic organizations of these early visual areas from cortical anatomy with high accuracy (*38*). Within each ROI, we measured (i) quantitative and diffusion parameters across cortical depths and (ii) in the superficial white matter underneath each ROI. Gray and superficial white matter were analyzed separately because they differ in tissue composition and developmental trajectories (*24*, *39*). The cortical gray matter primarily contains neuronal cell bodies, dendrites, axons, and local synaptic connections, which undergo early developmental processes such as synaptogenesis and pruning (*7*, *9*). In contrast, the superficial white matter consists of short association fibers (*40*) that exhibit protracted myelination and structural refinement extending until adulthood (*41*, *42*). To determine the effects of congenital blindness on cortical microstructure, we employed linear mixed-effects models. In addition, we (iii) measured cortical thickness and (iv) examined its association with microstructural measures, surface area, cortical curvature, and gyrification by including cortical thickness as a covariate in the linear mixed-effects models.

### Microstructural Group Differences in the Early Visual Cortex

**Fig. 2.**
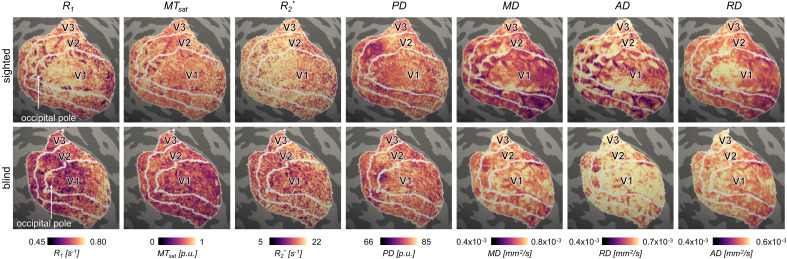
Exemplary quantitative multi-parameter maps (MPMs; *R_1_*, *MT_sat_*, R_2_^*^, and *PD*) and DTI-derived maps (*MD*, *RD*, and *AD*) of a blind and sighted participant at 3 T. Arrows (white) in the *R_1_* map indicate the location of the occipital pole. Borders between V1, V2 and V3 were defined by the Benson atlas (*38*).

Figure 2 shows exemplary multiparameter maps of a blind individual and a sighted control at 3 T.

#### Reduced R_2_^*^ and MT_sat_ in blind individuals

**Fig. 3.**
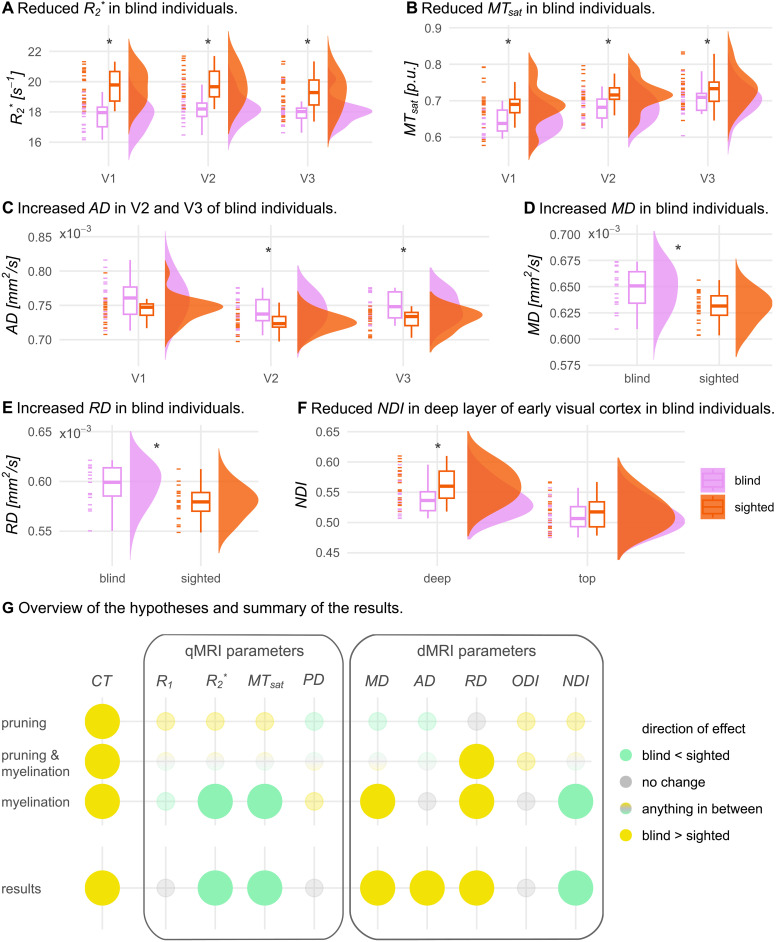
Lower iron and myelin content in the early visual cortex of congenitally blind individuals inferred from 3 T MRI. The distributions of parameter values are shown in raincloud plots and boxplots. Boxplots represent minimum, first quartile, median, third quartile, and maximum. Individual data points represent the mean parameter per participant. (**A** to **C**) The distribution of qMRI and dMRI parameter values per ROI (V1, V2, V3) and group (blind, sighted). Blind individuals had reduced R_2_^*^ (A) and reduced *MT_sat_* (B) in the early visual cortex, with the largest effects of group observed in V1 (V1 > V2 > V3). (C) Blind individuals had increased *AD* in V2 and V3. (**D** and **E**) The distributions of dMRI parameter values per group (blind, sighted). Blind individuals had increased *MD* (D) and *RD* (E) in the early visual cortex. (**F**) The distribution of neurite density (*NDI*) across cortical layers shows reduced values in the deep layer of the early visual cortex of blind individuals. (**G**) Summary of the results. The significant observations, shown as large, solid-colored circles, superimposed onto the pre-registered hypotheses, shown as smaller, more transparent circles, from Fig. 1. Overall, our results provide evidence for the notion of reduced myelination in the early visual cortex of congenitally blind individuals. *AD* = axial diffusivity, *MD* = mean diffusivity, *MT_sat_* = magnetization transfer saturation, *NDI* = neurite density index, *ODI* = orientation dispersion index, *PD* = proton density, *R_1_* = longitudinal relaxation rate, R_2_^*^ = effective transverse relaxation rate, *RD* = radial diffusivity. 7 T results are shown in fig. S2.

 At 3 T, blind individuals had lower R_2_^*^ in the early visual cortex than sighted controls. However, this effect varied by ROI, as indicated by a significant group × ROI interaction [Fig. 3A, *F*_(2, 88.60)_ = 3.58, *p* = 0.032]. As predicted by our pre-registered hypotheses, post-hoc tests conducted using approximate z-tests on the estimated marginal means of R_2_^*^ revealed the largest effects of group in V1 with estimated group differences per ROI in the range of −1.65 s^−1^ to −1.06 s^−1^ (all *p*s < 0.05, table S4). In addition, blind individuals had lower *MT_sat_* than sighted controls in all ROIs [Fig. 3B, group × ROI interaction: *F*_(2, 599.55)_ = 8.64, *p* < 0.001], with the largest effect in V1 (group differences per ROI: −0.072 p.u. to −0.031 p.u., *p*s < 0.05, table S5). No significant group differences were observed for *R_1_* and *PD* in the gray matter (all *p*s > 0.05). Taken together, these findings suggest reduced iron and myelin content in the early visual cortex of blind individuals compared to sighted controls.

The results at 7 T corroborated the results at 3 T. Blind individuals had lower R_2_^*^ compared to sighted controls [fig. S2; main effect of group: *F*_(1, 27.04)_ = 11.08, *p* = 0.003], with a mean estimated group difference of −2.80 ± 0.81 s^−1^ [mean ± standard error of the mean (s.e.m.); *df* = 28.6, *t-ratio* = −3.457, *p* = 0.002]. No significant group differences were observed for *R_1_* and *PD* in the gray matter (all *p*s > 0.05).

To assess whether the effects of reduced myelination were specific to the visual cortex rather than reflecting broader developmental differences, we conducted supplementary analyses of cortical thickness, R_2_^*^, and *MT_sat_* in primary sensory regions (A1, S1, and M1). These ROIs were defined using the Glasser Atlas (*43*). For each participant, we extracted mean cortical thickness, and mean R_2_^*^ and *MT_sat_* values sampled at mid-cortical depth within each ROI. None of these regions showed significant group differences (all *ps* > 0.05), suggesting that the microstructural changes in early visual cortex most likely arise from congenital blindness itself, rather than from a more general developmental effect.

#### Increased AD, MD, and RD in blind individuals

A significant group × ROI interaction was observed for *AD* [Fig. 3C, *F*_(2, 383.72)_ = 5.00, *p* = 0.007], suggesting that the effect of group varied across ROIs. Post-hoc tests revealed significantly higher *AD* in V2 and V3 of blind individuals (group differences per ROI: 1.959 × 10^−6^ mm^2^/s to 2.161 × 10^−6^ mm^2^/s, all *ps* < 0.05, table S6). We did not predict higher *AD* in blind individuals. In addition, we observed significant main effects of group for *MD* [*F*_(1, 39.29)_ = 9.66, *p* = 0.003] and *RD* [*F*_(1, 38.58)_ = 9.55, *p* = 0.004; Fig. 3, D and E]. On average, *MD* was increased by 1.680 × 10^−6^ ± 0.005 mm^2^/s (*df* = 40.4, *t-ratio* = 3.108, *p* = 0.003) and *RD* was increased by 1.740 × 10^−6^ ± 5.63 × 10^−3^ mm^2^/s (*df* = 40.1, *t-ratio* = 3.090, *p* = 0.004) in the blind compared to the sighted group. These findings provide further evidence for the notion of *lower myelination* of the early visual cortex in blind individuals.

#### Decreased neurite density in deep cortical layers in blind individuals

A significant group × cortical layer interaction was observed for *NDI* [Fig. 3F, *F*_(1, 83.18)_ = 23.40, *p* < 0.001], suggesting that the effect of group differed across cortical layers. Post-hoc tests revealed significantly lower *NDI* in the deep cortical layer (−0.02563 ± 0.00864, *df* = 39.7, *t-ratio* = −2.967, *p* = 0.01) in blind individuals, with no significant differences in the superficial cortical layer (−0.00805 ± 0.00830, *df* = 40.0, *t-ratio* = −0.970, *p* = 0.338). No significant group differences were observed in *ODI*. These findings support the notion of *lower myelination* of the early visual cortex in blind individuals.

### Multivariate patterns of cortical microstructure in early visual cortex

To identify dominant multivariate patterns across qMRI and dMRI measures, we applied principal component analysis (PCA) to a subject-level region × feature matrix comprising nine metrics of microstructural features: *MT_sat_*, *PD*, *R_1_*, R_2_^*^, *AD*, *MD*, *RD*, *NDI*, and *ODI*. PCA was performed on z-scored microstructural feature values across early visual regions (V1 – V3) for each participant.

**Fig. 4.**
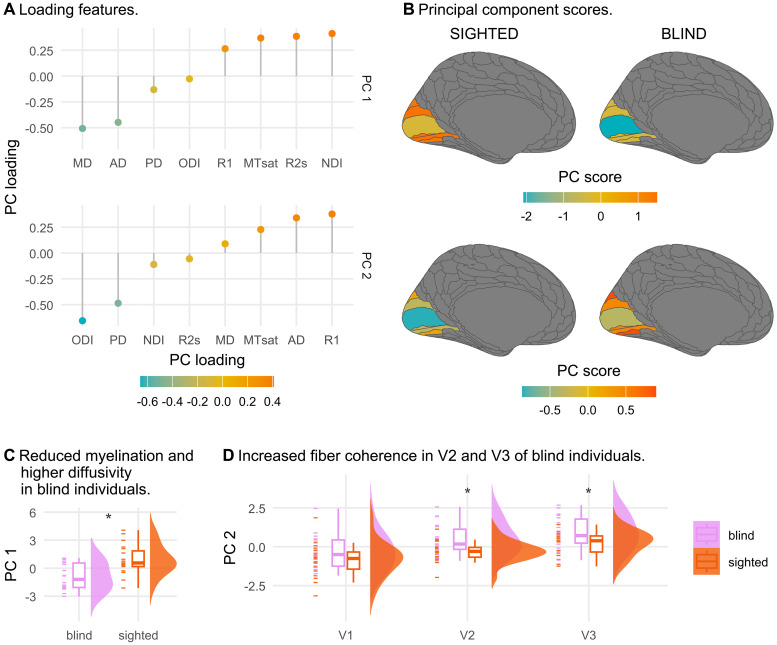
Altered microstructural profiles in the early visual cortex of blind individuals. (**A**) Principal component analysis (PCA) was applied to cortical microstructural metrics to identify orthogonal axes of maximal variance across the cortex. Loadings for the first two principal components (PCs) are shown (note the different order of labels on the horizontal axes). *PC1* likely reflects a “myelo-neurite density” axis: high positive loadings are associated with highly myelinated and structurally refined tissue (e.g., high *MT_sat_* and *R_1_*, low *RD* and *MD*), whereas low negative loadings indicate more isotropic, less myelinated tissue. *PC2* appears to index fiber orientation coherence, with higher scores linked to lower *ODI* values, indicating more coherently organized fibers. (**B**) Mean PC scores shown for each group in V1, V2, and V3. (**C**) Blind individuals exhibit significantly lower *PC1* scores, consistent with reduced myelination in early visual cortex. The plotted values represent the main effect of group using PC1 scores averaged across V1, V2, and V3. (**D**) Blind individuals show significantly higher *PC2* scores in V2 and V3, suggestive of increased fiber orientation coherence. Raincloud plots and boxplots depict the distribution of participant-averaged PCA scores. Boxplots indicate the minimum, first quartile, median, third quartile, and maximum. Individual dots represent mean values per participant. *AD* = axial diffusivity, *MD* = mean diffusivity, *MT_sat_* = magnetization transfer saturation, *NDI* = neurite density index, *ODI* = orientation dispersion index, *PD* = proton density, *R*_*1*_ = longitudinal relaxation rate, R_2_^*^ = effective transverse relaxation rate, *RD* = radial diffusivity.

The first principal component (*PC1*) accounted for 45.0% of the total variance and represented a dominant axis of microstructural variation across individuals. *PC1* exhibited strong positive loadings from myelin-sensitive measures (*MT_sat_*, *R_1_*, R_2_^*^) and neurite density (*NDI*), and strong negative loadings from diffusivity measures (*AD*, *MD*, *RD*) (Fig. 4A). This suggests a gradient from dense and highly myelinated tissue to more isotropic, less organized tissue. *PC2* explained an additional 16.5% of the variance and was characterized by negative loadings from *ODI* and *PD*, and positive loadings from *R_1_* and *AD*, potentially reflecting interindividual differences in fiber orientation coherence and microstructural complexity. PC3 accounted for 15.3% of the variance and was defined by positive loadings on *PD* and *MT_sat_* and negative loadings on *NDI* and *ODI*, indicating a more complex interplay between water content, myelin content, and neurite architecture, and its effects on MRI contrast parameters. Together, the first three components explained approximately 77% of the total variance, suggesting that a relatively small number of multivariate patterns capture most of the variability across qMRI and dMRI measures.

For *PC1*, we observed a significant main effect of group [*F*_(1, 37.40)_ = 11.95, *p* = 0.001]. Blind individuals had reduced scores relative to sighted controls (group difference = −1.7 ± 0.492; *df* = 37.8, *t-ratio* = −3.457, *p* = 0.001), suggesting lower myelin-related signal (*MT_sat_*, *NDI*, R_2_^*^, *R_1_*) and higher diffusivity (*RD*, *MD*, *AD*) in blind individuals. This finding is consistent with our univariate analyses and supports the notion that congenital blindness leads to *reduced intracortical myelination*. For *PC2*, we observed a significant group × ROI interaction [*F*_(1, 206.72)_ = 6.90, *p* = 0.009]. Post-hoc tests revealed increased *PC2* scores in blind individuals in V2 (group difference = 0.838 ± 0.326; *df* = 65.3, *t- ratio* = 2.569, *p* = 0.038) and V3 (group difference = 0.932 ± 0.389; *df* = 107.7, *t-ratio* = 2.396, *p* = 0.038), with no significant group differences in V1 (*P* > 0.05). This finding could reflect increased fiber orientation coherence and/or reduced dendritic complexity in blind individuals. For PC3, we did not observe any main effects or interactions involving the factor group.

### Microstructural Group Differences in the Superficial White Matter underneath the Early Visual Cortex

#### Reduced R_2_^*^ in blind individuals

**Fig. 5.**
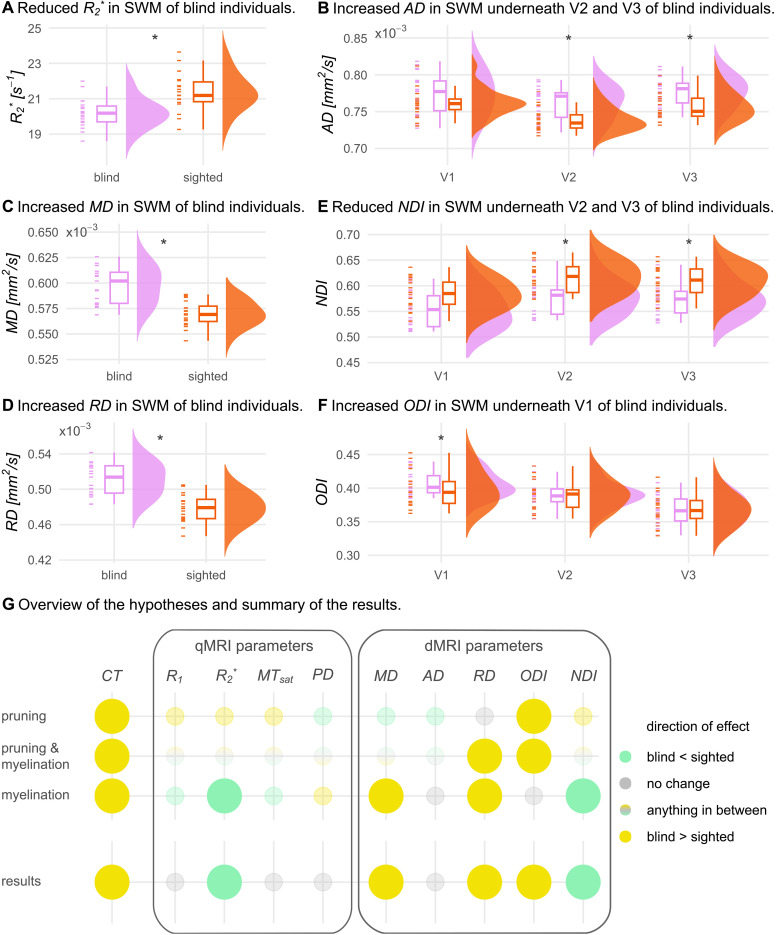
Lower iron and myelin content in the superficial white matter underneath the early visual cortex of congenitally blind individuals inferred from 3 T MRI. The distributions of parameter values are shown in raincloud plots and boxplots. Boxplots represent minimum, first quartile, median, third quartile, and maximum. Individual data points represent the mean parameter per participant. (**A**, **C**, and **D**) The distribution of qMRI and dMRI parameter values per group (blind, sighted) and (**B**, **E**, and **F**) the distribution of dMRI parameter values per ROI (V1, V2, V3) and group (blind, sighted). Blind individuals had reduced R_2_^*^ (A) as well as increased *MD* (C) and increased *RD* (E). Blind individuals had increased *AD* (B) and reduced *NDI* (E) in V2 and V3 and increased *ODI* (F) in V1. (G) Summary of the results. The significant observations, shown as large, solid-colored circles, superimposed onto the pre-registered hypotheses, shown as smaller, more transparent circles, from Fig. 1. Overall, our results provide evidence for the notion of reduced myelination in the early visual cortex of congenitally blind individuals. *AD* = axial diffusivity, *MD* = mean diffusivity, *MT_sat_* = magnetization transfer saturation, *NDI* = neurite density index, *ODI* = orientation dispersion index, *PD* = proton density, *R_1_* = longitudinal relaxation rate, R_2_^*^ = effective transverse relaxation rate, *RD* = radial diffusivity. 7 T results are shown in fig. S3.

A significant main effect of group [*F*_(1, 41.87)_ = 9.97, *p* = 0.003], indicated reduced R_2_^*^ in the SWM of blind individuals compared to sighted controls at 3 T (Fig. 5A). On average, R_2_^*^ in the SWM was reduced by −0.96 ± 0.304 s^−1^ (*df* = 41.7, *t-ratio* = −3.158, *p* = 0.003) in the blind compared to the sighted group. This finding is consistent with the notion of *lower myelination and lower iron content* in the early visual cortex of blind individuals. No significant group differences for *MT_sat_*, *R_1_*, or *PD* were observed in the SWM.

The results at 7 T corroborated the results at 3 T. Blind individuals had lower R_2_^*^ values compared to sighted controls [fig. S3; main effect of group: *F*_(1, 28.07)_ = 7.94, *p* = 0.009] with an estimated group difference of −2.04 ± 0.728 s^−1^ (*df* = 27.9, *t-ratio* = −2.808, *p* = 0.009). No significant group differences were observed for *R_1_* and *PD* in the superficial white matter (all *p*s > 0.05).

#### Increased AD, MD and RD in blind individuals

*AD* was increased in blind individuals compared to sighted controls. The effect of group varied across ROIs, as indicated by a significant group × ROI interaction [Fig. 5B, *F*_(2, 204.74)_ = 10.23, *p* < 0.001]. Post-hoc tests revealed that the effect of group was significant in the SWM underneath V2 and V3 (group difference per ROI: 2.599 × 10^−6^ mm^2^/s to 2.684 × 10^−6^ mm^2^/s, all *ps* < 0.05, table S7). Similar to the cortex, we did not predict higher *AD* in the SWM of blind individuals (Fig. 1). *MD* and *RD* were also increased in blind individuals compared to sighted controls [Fig. 5, C and D, main effect of group for *MD*: *F*_(1, 43.10)_ = 30.34, *p <* 0.001, and for *RD*: *F*(1, 41.66) = 38.39, *p* < 0.001]. On average, *MD* was increased by 2.7 × 10^−6^ ± 0.49 × 10^−6^ mm^2^/s (*df* = 43.1, *t-ratio* = 5.507, *p* < 0.001) and *RD* by 3.29 × 10^−6^ ± 0.531 × 10^−6^ mm^2^/s (*df* = 42.3, *t-ratio* = 6.195, *p* < 0.001) in the blind compared to the sighted group. These findings further support the notion that myelination is reduced in the SWM of blind individuals.

#### Reduced NDI and increased ODI in blind individuals

*NDI* was significantly reduced in the SWM of blind individuals. The effect of group varied across ROIs, as indicated by a significant group × ROI interaction [Fig. 5E, *F*_(2, 195.37)_ = 3.32, *p* = 0.038]. Post-hoc tests revealed that the effect of group was only significant in the SWM underneath V2 and V3 (group differences: −0.045 to −0.025, all *p*s < 0.05, table S8). This finding further supports the notion of *reduced myelination* in the SWM underlying the early visual cortex of blind individuals. In addition, blind individuals had a significantly higher *ODI* in the SWM. The effect of group varied across ROIs, as indicated by a significant group × ROI interaction [Fig. 5F, *F*_(2, 200.00)_ = 6.60, *p* = 0.002]. Post-hoc tests revealed that the effect of group was only significant in the SWM underneath V1 (0.02476 ± 0.00764, *df* = 90.2, *t-ratio* = 3.241, *p* = 0.005, all other *p*s > 0.05).

### Multivariate patterns of SWM microstructure underneath the early visual cortex

**Fig. 6.**
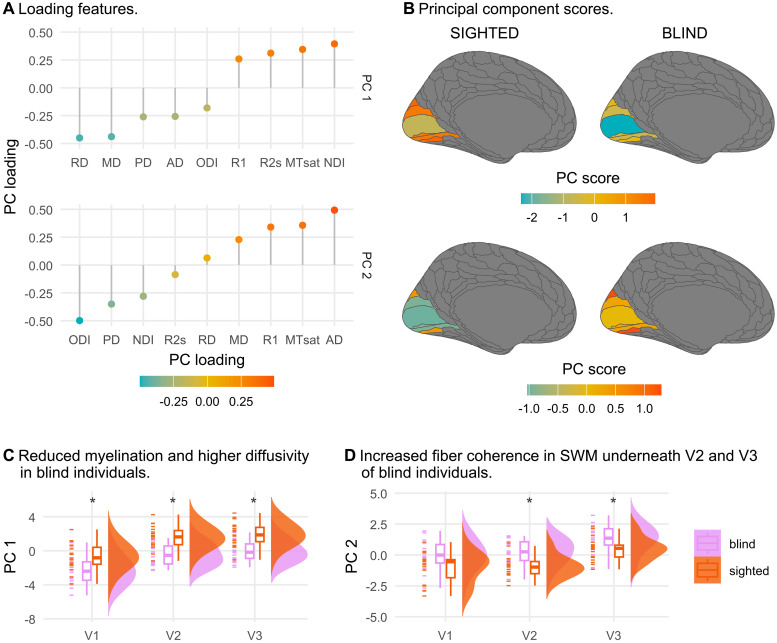
Altered microstructural profiles in the superficial white matter (SWM) underlying early visual cortex in blind individuals. (**A**) Principal component analysis (PCA) was applied to SWM microstructural metrics to identify orthogonal axes capturing the greatest variance across regions. Loadings for the first two principal components (PCs) are shown (note the different order of labels on the horizontal axes). *PC1* likely represents a “myelo-neurite density” axis, with high positive loadings associated with dense, highly myelinated tissue (e.g., high *MT_sat_*, *R_1_*, and *NDI*; low *RD* and *MD*), and negative loadings indicative of more isotropic, less myelinated tissue. *PC2* appears to capture fiber orientation coherence, with higher scores linked to lower *ODI* values, suggesting more coherent fiber organization. [(**B**), upper panel (**C**)] Blind individuals show significantly lower *PC1* scores across all regions of interest (ROIs), with the largest effects observed in V2 and V3, consistent with reduced myelination of the SWM underneath the early visual cortex. [(B), lower panel (**D**)] Blind individuals exhibit significantly higher *PC2* scores in V2 and V3, suggesting increased fiber orientation coherence. Raincloud plots and boxplots illustrate the distribution of PCA scores averaged per participant. Boxplots display the minimum, first quartile, median, third quartile, and maximum. Individual points represent mean values per participant. *AD* = axial diffusivity, *MD* = mean diffusivity, *MT_sat_* = magnetization transfer saturation, *NDI* = neurite density index, *ODI* = orientation dispersion index, *PD* = proton density, *R_1_* = longitudinal relaxation rate, R_2_^*^ = effective transverse relaxation rate, *RD* = radial diffusivity.

The first principal component (*PC1*) accounted for 46.5% of the total variance and represented a dominant axis of microstructural variation across individuals. Similarly to what we observed in the gray matter, *PC1* exhibited strong positive loadings from myelin-sensitive measures (*MT_sat_*, *R_1_*, R_2_^*^) and neurite density (*NDI*), and strong negative loadings from diffusivity measures (*AD*, *MD*, *RD*) and *PD* (Fig. 6A). *PC1* likely represents a “myelo-neurite density” axis, where higher values indicate increased myelination and neurite density, and lower values reflect reduced myelin content and increased diffusivity. *PC2* explained an additional 24.4% of the variance and was characterized by negative loadings from *ODI* and *PD*, and positive loadings from myelin sensitive measures like *R_1_* and *MT_sat_*, as well as *AD*. High positive loadings in this component may reflect more organized, coherent fibers, whereas high negative loadings may reflect more dispersed or complex fiber orientation patterns. PC3 accounted for 10.3% of the variance and was defined by positive loadings on *PD* and negative loadings on *R_1_*, R_2_^*^ and *ODI*, indicating a more complex interplay between water content, myelin content, and neurite architecture. High positive loadings in this component may reflect more free water and less myelin, whereas high negative loadings may reflect more myelinated, organized tissue. Together, the first three components explained approximately 81.3% of the total variance, suggesting that a relatively small number of multivariate patterns capture most of the variability across qMRI and dMRI measures in the SWM.

For *PC1*, there was a significant group × ROI interaction [Fig. 6C; *F*_(2, 182.01)_ = 3.29, *p* = 0.04]. Across all three ROIs, blind individuals exhibited significantly lower *PC1* scores compared to sighted controls, with the largest effects observed in V2 and V3 (group differences ranging from −1.37 to −2.15; all *ps* < 0.05; table S9). These reductions in *PC1* score likely reflect decreased myelin-sensitive signal (e.g., *MT_sat_*, *R_1_*) and increased diffusivity (e.g., *MD*, *RD*) in blind individuals. These findings support the hypothesis that congenital blindness leads to *reduced myelination* in the SWM underneath the early visual cortex. For *PC2*, we also observed a significant group × ROI interaction [Fig. 6D; *F*_(1, 193.28)_ = 5.36, *p* = 0.005]. Post-hoc tests revealed significantly increased *PC2* scores in blind individuals in V2 (group difference = 1.293 ± 0.359; *df* = 73.9, *t-ratio* = 3.598, *p* = 0.002) and V3 (group difference = 1.019 ± 0.44; *df* = 123.0, *t-ratio* = 2.319, *p* = 0.044), with no significant group difference in V1 (*P* > 0.05). These results possibly reflect increased fiber orientation coherence and reduced dendritic complexity in blind individuals. Since orientation coherence is expected to increase through pruning of redundant or misaligned dendritic and axonal processes (*44*), these findings suggest that congenital blindness may not impair, and may even enhance, certain aspects of microstructural refinement in early visual areas. For PC3, we did not observe any significant main effects or interactions involving the factor group.

### Associations of Cortical Thickness with Microstructure and Surface Area

#### Increased cortical thickness in V2 of blind individuals

**Fig. 7.**
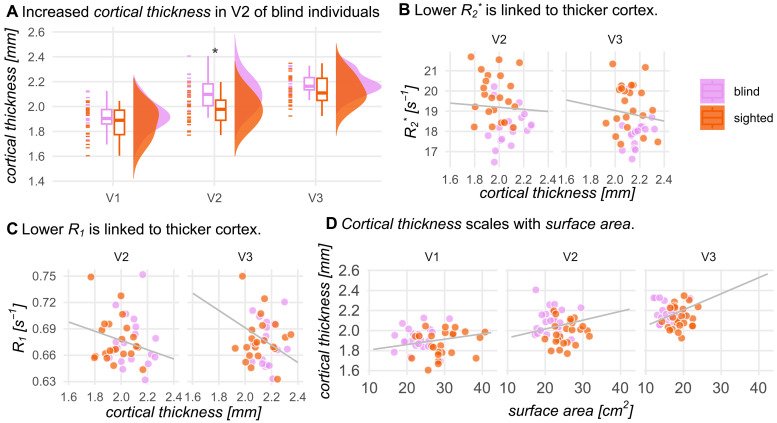
Lower R_2_^*^ and *MT_sat_* in the cortical gray matter are associated with thicker early visual cortex. (**A**) Increased cortical thickness in V2 of blind individuals. Cortical thickness is shown for each ROI (V1, V2, V3) and group (blind, sighted) in raincloud plots and boxplots. The boxplots represent minimum, quartile 1, median, quartile 3, and maximum cortical thicknesses. Individual data points represent the mean cortical thickness for each participant and region. (**B**) Negative association between R_2_^*^ and cortical thickness in V2 and V3. (**C**) Negative association between *R_1_* and cortical thickness in V2 and V3. (**D**) Positive association between cortical thickness and surface area. (B to D) Line plots of the linear fit relating cortical thickness and surface area in each ROI. Individual data points represent the mean cortical thickness plotted against the mean surface area for each participant and ROI. Note that surface area is shown in its original (non-centered) form to aid comparability with previous studies. However, statistical analyses were conducted using mean-centered surface area values. *R_1_* = longitudinal relaxation rate, R_2_^*^ = effective transverse relaxation rate.

Consistent with previous studies, early visual cortices were thicker in blind individuals compared to sighted controls. However, this effect varied by region as shown by a significant group × ROI interaction [Fig. 7A, *F*_(2, 231.00)_ = 6.54, *p* = 0.002]. Post-hoc tests revealed a significant difference only in V2, where the cortex was on average 0.126 ± 0.033 mm thicker in blind individuals (*df* = 62.5, *t-ratio* = 3.849, *p* < 0.001). No significant group differences were observed in V1 and V3 (all other *p*s > 0.05, table S10). See Supplementary Materials for analyses of surface area, curvature, and gyrification.

#### Lower iron and myelin content in the gray and superficial white matter are associated with thicker V2 and V3

The analysis of the 3 T data revealed a negative association between cortical thickness and R_2_^*^. This association varied across ROIs [Fig. 7B, ROI × cortical thickness interaction: *F*_(2, 100.84)_ = 7.39, *p* = 0.001] and was significant only in V2 and V3 (slope in V1 = 0.057 s^−1^/mm, CI = [−1.060, 1.178] s^−1^/mm; slope in V2 = −1.626 s^−1^/mm, CI = [−2.670, −0.578] s^−1^/mm; slope in V3 = −2.146 s^−1^/mm, CI = [−3.300, −0.996] s^−1^/mm). This finding suggests that lower cortical iron content is associated with thicker early visual cortex. In addition, we observed a negative association between cortical thickness and *R_1_*, which also varied across ROIs [Fig. 7C, ROI × cortical thickness interaction: *F*_(2, 667.27)_ = 12.07, *p* < 0.001] and was also only significant in V2 and V3 (slope in V1 = −0.0293 s^−1^/mm, CI = [−0.071, 0.0123] s^−1^/mm; slope in V2 = −0.0698 s^−1^/mm, CI = [−0.113, −0.0266] s^−1^/mm; slope in V3 = −0.112 s^−1^/mm, CI = [−0.16, −0.0637] s^−1^/mm). Similarly, *R_1_* in the SWM and cortical thickness were negatively associated in V3 [fig. S6C, ROI × cortical thickness interaction: *F*_(2, 197.14)_ = 6.12, *p* = 0.003, slope V3 = −0.0800 s^−1^/mm, CI = [−0.142, −0.0182] s^−1^/mm]. Slopes for the associations in V1 and V2 were not different from 0. Together, these findings suggest that lower myelin content of the cortical gray and superficial white matter is associated with thicker early visual cortex. This finding is consistent with previous reports (*10*). See Supplementary Materials for associations between other qMRI and dMRI parameters and cortical thickness.

The analysis of the 7 T data revealed similar results: there was a significant interaction between group, ROI, and cortical thickness on *R_1_* values [group × ROI × cortical thickness interaction: *F*_(2, 452.22)_ = 3.55, *p* = 0.029]. In V1 of blind individuals, cortical thickness and *R_1_* were negatively associated (slope = −0.083 s^−1^/mm, CI = [−0.1551, −0.01095] s^−1^/mm). In contrast, this association was not significant in V1 of sighted controls (slope = −0.0182 s^−1^/mm, CI = [−0.0779, 0.04148] s^−1^/mm). Conversely, in V3 of sighted individuals, there was a significant negative association between cortical thickness and *R_1_* (slope = −0.0693 s^−1^/mm, CI = [−0.1306, −0.00789] s^−1^/mm), whereas this relationship was not significant in blind individuals (slope = −0.041 s^−1^/mm, CI = [−0.1228, 0.04087] s^−1^/mm). Other slopes did not significantly differ from zero, as their confidence intervals included zero.

#### Principal components of cortical microstructure show distinct associations with cortical thickness

We observed a positive association between *PC1* scores and cortical thickness in V1 [ROI x thickness interaction: *F*_(2, 174.81)_ = 10.32, *p* < 0.001; slope in V1 = 3.8629 mm^−1^, CI = [2.12, 5.61] mm^−1^; other slopes not different from 0], suggesting that thicker V1 has a microstructural profile associated with higher *PC1* scores. Given that the univariate analyses revealed negative associations between diffusivity measures (*RD*, *MD*, *AD*) and cortical thickness, and a negative association between *MT_sat_* and cortical thickness, the positive association between *PC1* and cortical thickness in V1 is likely driven by the diffusion-related loadings. For *PC2*, we observed a group × cortical thickness interaction [*F*_(1, 206.72)_ = 6.90, *p* = 0.00]: in blind individuals, higher *PC2* scores were associated with thinner cortex (slope = −2.06 mm^−1^, CI = [−3.857, −0.255] mm^−1^), whereas this relationship was absent in sighted controls (slope = 1.4 mm^−1^, CI = [−0.507, 3.31] mm^−1^), suggesting that in blind individuals increased orientation coherence is associated with thinner cortex. This could in turn suggest that in blind individuals with thicker cortices, the cortices are less pruned. For PC3, we observed a positive relationship between PC3 scores and cortical thickness [*F*_(1, 195.80)_ = 11.03, *p* = 0.001; slope: 1.56 mm^−1^, CI = [0.63, 2.49] mm^−1^]. PC3 increases with less myelin, fewer and/or diffuse neurites, and higher water content (*PD*).

#### Distinct associations between superficial white matter microstructure and cortical thickness in blind individuals

We observed a significant group × ROI × cortical thickness interaction on *PC1* scores in the superficial white matter [*F*_(2, 175.28)_ = 3.38, *p* = 0.036], indicating that the relationship between cortical thickness and *PC1* varied by group and ROI. There was a positive association between cortical thickness and *PC1* in V1 of blind individuals (slope = 6.865 mm^−1^, CI = [4.502, 9.23] mm^−1^) and sighted controls (slope = 2.728 mm^−1^, CI = [0.555, 4.90] mm^−1^). The comparisons of the slopes between the two groups revealed that the slope in blind individuals was significantly steeper than in sighted individuals (4.137 ± 1.63 mm^−1^, df = 188, t-ratio = 2.542, p = 0.012). No significant associations were observed in the other ROIs. As in the gray matter, this positive association between *PC1* and cortical thickness in V1 is likely driven primarily by diffusion-related loadings. For *PC2*, a significant group × cortical thickness interaction was observed [*F*_(1, 198.91)_ = 9.42, *p* = 0.002]. In sighted individuals, cortical thickness was positively associated with *PC2* scores (slope = 3.05 mm^−1^, CI = [0.83, 5.27] mm^−1^), suggesting that reduced fiber coherence in SWM is linked to thinner cortex. In contrast, no such association was observed in the blind group (slope = −1.68 mm^−1^, CI = [−3.81, 0.45] mm^−1^). For PC3, we found a significant main effect of cortical thickness across groups [*F*_(1, 195.09)_ = 15.42, *p* < 0.001], with higher PC3 scores associated with greater cortical thickness (slope = 1.56 mm^−1^, CI = [0.77, 2.34] mm^−1^). Given that PC3 is positively associated with free water content (*PD*) and negatively with myelination and neurite density, this result aligns with the interpretation that thicker cortex is associated with less myelinated superficial white matter.

#### Cortical thickness scales with surface area

To test whether cortical thickness was associated with cortical curvature, surface area or local gyrification, we ran three separate linear mixed model (LMM) analyses assessing the effect of group and ROI on cortical thickness with curvature, surface area, or local gyrification as a covariate. We observed a positive association between cortical thickness and surface area. This effect varied by ROI [Fig. 7D, ROI × surface area interaction: *F*_(2, 91.88)_ = 4.50, *p* = 0.014], with the largest effects observed in V3 (slope in V1 = 5.34 × 10^−3^ mm/cm^2^, CI = [4.01 × 10^−4^, 1.03 × 10^−2^] mm/cm^2^; slope in V2 = 8.84 × 10^−3^ mm/cm^2^, CI = [2.16 × 10^−4^, 1.55 × 10^−2^] mm/cm^2^; slope in V3 = 1.61 × 10^−2^ mm/cm^2^, CI = [9.61 × 10^−3^, 2.25 × 10^−2^] mm/cm^2^). Thus, the thicker the cortex, the larger the surface area. This finding contradicts the notion that the cortex stretches and thins during development (*10*). No significant associations were observed between cortical thickness and mean cortical curvature or local gyrification (all *ps* > 0.05). See Supplementary Material for analyses of surface area, and how it relates to cortical curvature and gyrification.

## DISCUSSION

Using non-invasive in vivo histology with MRI (hMRI), we examined how visual experience—or the lack thereof—shapes the microstructure of the early visual cortex in humans. Our findings suggest that congenital blindness reduces myelination and oligodendrogenesis. This is supported by converging evidence from multiple complementary MRI-derived microstructural markers, including reduced R_2_^*^, *MT_sat_*, and neurite density index, and increased diffusivity (*MD*, *AD*, and *RD*). Taken together, these results highlight the critical role of visual experience in regulating cortical myelination and challenge the long-standing view that the increased cortical thickness of the visual cortex observed in blind individuals directly results from reduced pruning (*13*, *14*).

This study provides multi-modal in vivo evidence in humans consistent with reduced myelination in the early visual cortex of congenitally blind individuals. High-resolution data from 7 T and 3 T Connectom MRI scanners enabled the mapping of tissue microstructure at sub-millimeter scale, revealing several key patterns. First, *MT_sat_*, which is a validated proxy for myelin content (*45*–*48*), was reduced in the gray matter of blind individuals. Second, diffusivity metrics (*RD* and *MD*) were increased in the gray and superficial white matter of blind individuals. Animal studies using the dysmyelinating shiverer and cuprizone mouse models of reversible demyelination have shown that demyelination leads to an increase in *RD* (*30*, *31*). Moreover, a recent study in infants revealed a sharp decrease in *MD* during the first six months of life, accompanied by a marked increase in myelin-related markers (*29*), further linking diffusivity reductions to developmental myelination. Third, neurite density index, which is strongly linked to myelin content (*49*, *50*), was reduced in the gray and superficial white matter of blind individuals, further supporting the notion of reduced myelination.

To summarize the microstructural patterns related to congenital blindness, we employed a data-driven analysis that revealed a principal component reflecting a gradient of myelination-related features: higher scores were associated with increased MRI markers of myelination, higher neurite density, and lower diffusivity. Blind individuals showed significantly lower scores on this myelination axis, reinforcing the conclusions drawn from our univariate analyses and offering converging evidence for reduced myelination in the early visual cortex. Our findings align with animal studies showing that dark rearing can reduce developmental myelination of the optic nerve (*51*), while premature eye-opening can accelerate it (*52*), suggesting a critical role of visual experience in myelin development. Our study demonstrates these effects in the human brain, providing direct evidence that visual experience shapes cortical myelination during development.

While our study cannot directly separate developmental from adult-onset effects, prior research indicates that increased cortical thickness in the early visual cortex of blind individuals is already established early in life (*15*) and is not significantly influenced by late-onset blindness (*13*, *53*). Given that myelination in the visual cortex mainly develops in early childhood (*29*, *54*), our findings most likely reflect early developmental consequences of congenital blindness.

We observed few layer-specific effects, consistent with a global rather than layer-selective reduction in myelination. Pioneering work by Trampel *et al*. (*55*) demonstrated the presence of the stria of Gennari in both congenitally blind and sighted participants. Comparisons of signal intensity profiles across layers revealed strikingly similar patterns in both groups, including a pronounced dip at the approximate location of the stria of Gennari, suggesting preserved laminar organization. Taken together, the findings of Trampel *et al*. (*55*) and our own indicate that myelination of both radial and tangential fibers is proportionally reduced in congenitally blind individuals.

Reduced myelination may contribute to apparent increases in cortical thickness in blindness via altered MRI contrast at the gray–white matter boundary, which is used in MRI-based brain tissue segmentation. We observed negative associations between measures of iron and myelin (R_2_^*^, *R_1_*) content and cortical thickness in V2 and V3. Thus, the thinner the cortex, the higher the relative iron and myelin content of the underlying tissue. This is in line with results of Natu *et al*. (*10*) and the notion that thinner appearing cortex is more myelinated.

Congenital blindness may not only reduce myelination but also affect stages of oligodendrogenesis preceding myelination. Our findings of significantly reduced R_2_^*^ within the gray and superficial white matter in congenitally blind individuals support this hypothesis. Previous studies using advanced histology and *postmortem* MRI have identified the iron in oligodendrocyte bodies and related fibers as the primary source of R_2_^*^ contrast in the superficial white matter (*24*, *56*). Thus, the lower R_2_^*^ observed in congenitally blind individuals is consistent with lower counts of iron-rich mature oligodendrocytes or related fibers, highlighting the crucial role of visual experience in regulating oligodendrogenesis. It should be noted that oligodendrogenesis was not histologically measured in this study, and our interpretation is inferential, based on the known biophysical underpinnings of R_2_^*^. However, support for our interpretation comes from animal models that have shown that sensory deprivation dramatically reduces proliferation of oligodendrocyte precursor cells (*57*), whereas neuronal activity promotes it (*58*). Together, these observations suggest that visual experience is crucial in regulating developmental oligodendrogenesis and myelination.

Our findings suggest that changes in myelination may have long-term consequences for cortical circuit maturation and plasticity. Developmental myelination has been proposed to stabilize neuronal circuits and to limit certain forms of experience-dependent plasticity in adulthood (*59*, *60*). In mice, the myelination within the input layer of the cortex coincides with the closure of the critical period for neuronal plasticity (*60*). Moreover, absence of adolescent oligodendrogenesis, and the associated lack of myelination, extends plasticity into adulthood (*61*). Taken together with our results, these findings establish a critical role for oligodendrocytes and myelination in the maturation and stabilization of cortical circuits (*61*). Increased cortical excitability and functional reorganization observed in blind individuals may, in part, arise from these delayed maturational processes. However, the exact relationship between microstructural maturation (e.g., myelination), cortical thickness, and functional plasticity remains unresolved.

A longstanding hypothesis has attributed increased cortical thickness in blindness to disrupted pruning (*13*, *14*). Pruning eliminates redundant synapses and axons during development, and reductions in this process should produce a broader pattern of changes across MRI markers, including higher *R_1_*, R_2_^*^, *MT_sat_*, and neurite density, and lower *PD*, *AD*, and *MD*. We did not observe such a pattern. We note that several diffusion measures, such as *RD*, *MD*, and *NDI*, share some biological sensitivity with the MPM markers of myelination, and should therefore be considered complementary rather than fully independent lines of evidence. However, *AD* and *ODI* in gray matter are less directly coupled to myelin content, and their convergence with the MPM findings reflects sensitivity to distinct microstructural properties, strengthening the interpretation based on an overall pattern.

In addition to reduced R_2_^*^ and increased diffusivity measures, which point to reduced myelination in congenitally blind individuals, we observed increased orientation dispersion index in the superficial white matter beneath V1, indicating more heterogeneous fiber orientations (*50*). The heterogeneity of cortical fiber orientations depends on the relative proportions of tangential fibers to radial fibers and is typically highest in regions with prominent Baillarger bands (*33*). The increase in orientation dispersion observed in blind individuals could therefore arise from a reduction in the radial projections (e.g., geniculo-cortical) and/or an increase in tangential fibers. Although reduced pruning could, in principle, increase local fiber complexity, previous research has reported a reduction in geniculo-cortical projections in blind individuals (*62*). Thus, the increase in orientation dispersion is more consistent with a loss of radial input than with reduced pruning.

Our data-driven approach revealed an orientation dispersion versus fiber alignment axis along which blind individuals showed higher scores than sighted controls in V2 and V3. This finding could reflect increased fiber orientation coherence and/or reduced dendritic complexity in blind individuals. Since orientation coherence is expected to increase through pruning of redundant or misaligned dendritic and axonal processes (*44*), these findings suggest that congenital blindness may not impair—and may even enhance—certain aspects of microstructural refinement via pruning in these areas.

Reduced pruning and reduced myelination are not mutually exclusive. Pruning and myelination occur during partially overlapping developmental time windows (*9*) and congenital blindness could influence both. Activity-dependent myelination preferentially targets stable, consistently active axons (*63*); reduced pruning in congenital blindness could generate disorganized activity patterns that may fail to provide sufficient activity-dependent signals required for oligodendrocyte differentiation and maturation, thereby reducing myelination. Thus, decreased myelination observed in blind individuals may partly reflect downstream consequences of altered activity-dependent pruning.

Nevertheless, the rationale of our approach was that reduced pruning and reduced myelination are expected to exert opposing effects on tissue microstructure and could partially offset one another in MRI measures: reduced pruning could increase the density of synapses, dendrites, and unmyelinated axons, thus, increasing macromolecular content and restricting water diffusion, whereas reduced myelination could reduce myelin-associated macromolecules and increase extracellular water content. The net effect on MRI parameters thus depends on the relative magnitudes of these competing influences. Our observation of reduced *MT_sat_*, R_2_^*^, and *NDI*, together with increased diffusivity measures, suggests that reduced myelination dominates over potential alterations in pruning in shaping the overall tissue composition. So far, the longstanding view that pruning is disrupted in congenitally blind individuals is only supported by an extrapolation of animal research results to MRI-based cortical thickness in humans (*13*, *14*, *64*). Our multi-contrast data do not show the microstructural signature expected for reduced pruning; rather, the observed pattern is more consistent with reduced myelination.

Our results provide compelling evidence for altered microstructure in congenital blindness. However, they must be interpreted within the context of several methodological considerations. Although the qMRI and dMRI metrics are well established, they are indirect proxies of the underlying myelo- and cytoarchitecture, and no single metric maps uniquely to one specific microstructural feature such as myelin or iron content (For further details and critical discussion on contrast interpretation, see ref. *17* and Supplementary Materials). Since there is no simple one-to-one relationship between the MRI metrics and microstructural features, a multi-contrast approach was used to aid interpretation and detect proxy signatures of pruning and myelination (with pre-registered hypothesis as in Fig. 1).

Moreover, even at sub-millimeter image resolution, partial volume effects across cortical layers or the thin superficial white matter are not eliminated. We note that segmentation and definition of cortical layers may be affected by changes due to different visual experiences, which may in turn impact derived metrics. These methodological considerations are particularly relevant when evaluating the spatial and layer-specific distribution of the observed differences in qMRI and dMRI metrics. Most of our comparisons showed no evidence of layer-specific effects at the resolution of our three-compartment model, suggesting a global reduction in myelination within the early visual cortex of blind individuals. However, our approach involved spatial averaging (from 11 sampled cortical depths to three compartments for the qMRI data and from eight sampled cortical depths to two compartments for the dMRI data) and statistically complex models with four-way interactions, which may have limited our sensitivity to detect more subtle layer-specific differences.

We used the Benson atlas (*38*) to define early visual areas. While this atlas is anatomically grounded and retinotopically informed, it was developed using data from sighted individuals. The application of this atlas to blind individuals may introduce anatomical biases, given that visual experience affects the anatomy of early visual areas, including surface area, gyrification, curvature, and cortical thickness (*13*, *14*). However, the observed effects of blindness on cortical and superficial white matter microstructure were mostly consistent across all ROIs, suggesting that our conclusions are robust to the choice of atlas. Future studies could improve accuracy by using individualized parcellations based on functional connectivity or microstructural gradients (*43*). While these general limitations affect all non-invasive MRI studies, the presented data and analyses constitute a major advance in specificity, spatial resolution, and precision in the study of congenital blindness and its impact on cortical and superficial white matter development.

Finally, while our findings support a key role for reduced myelination and oligodendrogenesis in the early visual cortex of blind individuals, the link between structure and function remains complex. Animal studies have shown that disrupting adolescent oligodendrogenesis, and the associated delay in myelination, reduces inhibitory synaptic transmission (*61*), which is a key regulator of experience-dependent plasticity at the circuit level. In blind humans, an increased excitation–inhibition (E/I) ratio has been reported (*65*), potentially contributing to the heightened responses to non-visual stimuli observed in the visual cortex. However, human studies investigating the relationship between cortical thickness and fMRI blood-oxygenation-level dependent (BOLD) activation have generally found negative associations between the two (*66*, *67*), challenging the idea that increased thickness, and the associated decreased myelination, underlies heightened functional responses. Thus, the precise relationship between structural and microstructural changes (e.g., cortical thickness, myelin) and functional plasticity (e.g., BOLD activation, E/I balance) in blindness remains unresolved. Whether reduced myelination represents an independent effect of blindness, a downstream consequence of altered early circuit refinement, or a combination of both remains an open question. Resolving this issue will require longitudinal studies, particularly developmental investigations that incorporate histological validation. Future studies combining multimodal imaging (e.g., fMRI, MR spectroscopy, and qMRI) with computational modelling may also help clarify how changes in cortical (micro)structure relate to sensory reorganization and plasticity across development.

In summary, our findings demonstrate that a lack of visual input during development results in reduced iron and myelin content in the early visual cortex, as measured by sub-millimeter resolution quantitative and diffusion MRI. These findings highlight the crucial role of visual experience in regulating oligodendrogenesis and myelination. Although disrupted pruning has often been proposed as the main mechanism underlying cortical changes in blindness, we found only limited support for this notion. Given the established role of myelination in stabilizing cortical circuits and constraining plasticity, these results deepen our understanding of the mechanisms by which congenital blindness shapes brain development and plasticity in humans. Our results challenge the prevailing notion that pruning is the primary driver of structural changes observed in blind individuals and argue for a broader perspective that includes myelination as a central factor.

## MATERIALS AND METHODS

### Participant information

MRI data of 24 congenitally blind individuals (female/male: 10/14, mean age: 35.01 years, age range: 19.2–47.2 years, secondary/higher education = 11/13) and 24 sighted controls (female/male: 10/14, mean age: 34.32 years, age range: 22.11–48.7 years, secondary/higher education = 11/13) were included. No significant (at threshold *p* < 0.05) differences were observed in sex, age and education between the two groups. Neuroradiological inspection of the MRI data revealed incidental findings, which were addressed in accordance with institutional protocols and established ethical guidelines. No participants were excluded for this reason as the incidental findings were unrelated to the regions of interest (ROI) examined in this study. However, we excluded participants with missing structural MRI and/or images affected by motion artifacts. Details of the sample size for each analysis and the exclusion criteria are provided in tables S1 and S2.

For all congenitally blind participants, blindness was attributed to a peripheral origin and was not associated with any other sensory disabilities. To be included in the study, participants had to either be completely blind or, at most, have minimal light sensitivity. All sighted participants had normal or corrected-to-normal vision. None of the participants reported any history of psychiatric or neurological conditions.

All participants gave written informed consent for participation in the study. They received monetary compensation for their participation and were reimbursed for the travel costs. The study was approved by the local ethics committee of the Jagiellonian University (reference number: ke/63_2021) and the local ethics committee of the Medical Faculty of the University of Leipzig (reference number: 400/22-ek).

### Quantitative MRI

#### qMRI acquisition – 7 T

Data for estimating the MR relaxation parameters (*R_1_*, R_2_^*^, *PD*) were acquired using a 7 T whole-body MRI scanner (MAGNETOM Terra 7 T, Siemens Healthineers, Erlangen, Germany) equipped with SC72 body gradients (maximum gradient strength: 70 mT/m; maximum slew rate: 200 T/m/s) and an 8-channel transmit/32-channel receive RF head coil (Nova Medical, Wilmington, MA, USA).

Multi-parameter mapping (MPM) (*26*, *68*) was performed using a multi-echo variable flip angle protocol adapted from Vaculčiaková *et al*. (*25*). First, the vendor’s automatic B0 shimming was repeated three times. Then, calibration measurements of the radiofrequency (RF) transmit voltage were acquired and the vendor’s B1 shimming routine was used. This was followed by the acquisition of two multi-echo 3D fast low angle shot (FLASH) whole brain scans with T1- and *PD*-weighting (T1w, PDw) and 500 μm isotropic resolution using the following parameters: TR = 23 ms, TE = 3.06 ms to 15.66 ms in steps of 2.52 ms, flip angle (PDw/T1w) = 7°/22°, FOV = 176 × 224 × 256 mm^3^ (LR × AP × HF), readout bandwidth (rBW) = 445 Hz/pixel, and CAIPI (*69*) = 2 × 2 in both phase-encoding directions. Lastly, a B1+ map with 4 mm isotropic resolution was collected for flip angle correction using a 3D-AFI (*68*, *70*) protocol (flip angle = 55°, TR1/TR2 = 25 ms/125 ms, TE = 2.13 ms). During scanning, an optical tracking system was used for prospective motion correction (Kineticor, HI, USA). For motion detection, mouth guard assemblies with attached markers were custom-made for each participant by the Department of Cariology, Endodontology and Periodontology of the University of Leipzig Medical Center (*25*). The total scanning time of this MPM protocol was approximately 37 min.

#### qMRI acquisition – 3 T

Data for estimating the MR relaxation parameters (*R_1_*, R_2_^*^, *PD*) and *MT_sat_* were also acquired on a 3 T whole-body MR scanner (MAGNETOM Skyra CONNECTOM, Siemens Healthineers, Erlangen, Germany) equipped with a high-performance gradient system with 300 mT/m maximum gradient amplitude and a slew rate of 200 T/m/s. The vendor’s single-channel transmit RF body coil and a 32-channel receive RF head coil were used.

At the beginning of each scanning session, a B1+ map (4 mm isotropic resolution) was acquired for flip angle correction using a 3D-EPI spin echo-stimulated echo acquisition and corrected for susceptibility artefacts using a 2D-GRE fieldmap (*71*). Then, low resolution (4 mm isotropic) *PD*-weighted head and body coil-receive images were acquired before each contrast to correct for changes in receiver sensitivity due to motion (*72*). Whole-brain qMRI data were collected using T1w, PDw, and MT-weighted (MTw) multi-echo 3D gradient echo sequences (*26*) with 800 μm isotropic resolution using the following parameters: TR = 25 ms, TE = 2.40 ms to 18.64 ms (14 ms for MTw) in steps of 2.32 ms, flip angle (PDw and MTw) = 6°; flip angle (T1w) = 21°, FOV = 176 × 220 × 256 mm^3^ (LR × AP × HF), rBW = 488 Hz/px, and GRAPPA = 2 × 2 in both phase-encoding directions. MT-weighting was achieved with a 180° Gaussian pulse applied 2 kHz off-resonance. The total scanning time of this MPM protocol was approximately 28 min.

#### qMRI mapping

Quantitative parameter maps were computed from the acquired MPMs at 3 T and 7 T separately using the hMRI toolbox (*73*) (v0.2.4–335-geec6213, https://hmri-group.github.io/hMRI-toolbox/) implemented in SPM12. T1w, PDw, and MTw (only for the 3 T data) images from the MPM protocol were averaged across all echoes and the registration between all three contrasts was computed using SPM12. R_2_^*^ maps were calculated based on all the available echoes across all contrasts by log-linear weighted least squares (WLS) estimation (*74*). T1w and PDw images were extrapolated to TE = 0 to correct for R_2_^*^ weighting bias. The corrected images were then used to calculate *R_1_* and *PD* maps using a solution of the Ernst equation for short-TR dual flip angle measurements (*74*–*76*). *PD* maps were corrected for the receiver coil sensitivity profile using the unified segmentation method (*77*) and were calibrated to a mean of 69 percent units (p.u.) in the white matter (*73*, *78*). The *MT_sat_* maps were constructed using the procedure described in Helms *et al*. (*79*). *MT_sat_* is a semi-quantitative metric reflecting the percentage loss of observable steady state magnetization due to the MT pre-pulse and differs from the commonly used MT ratio by explicitly accounting for spatially varying T1 relaxation times and flip angles (*26*). The final qMRI maps (*R_1_*, R_2_^*^, *PD*, *MT_sat_*) were corrected for spatial distortions due to scanner gradient nonlinearity using the gradunwarp toolbox (*80*) (v1.2.1, https://github.com/Washington-University/gradunwarp) and spherical harmonic coefficients provided by the scanner manufacturer.

### Diffusion MRI

#### dMRI acquisition

The DWI protocol was adapted from Movahedian Attar *et al*. (*18*), slightly modified by reducing the spatial resolution (1.0 mm isotropic resolution) and adding multiband acceleration (MB factor 2) to accommodate time constraints. The DWI protocol enabled acquisition of a slab of 62 oblique near-axial slices, which was placed to cover V1, V2, and V3 in the early visual cortex in each participant. DWI were acquired on a 3 T Connectom MRI scanner with b-values of 800 s/mm^2^ and 1800 s/mm^2^, each acquired along 60 non-collinear diffusion-encoding directions. The diffusion-encoding directions were uniformly distributed over the whole sphere to optimize correction for eddy current (EC)-induced geometric distortions (*81*). A total of 30 reference non-diffusion-weighted images (b = 0 s/mm^2^) were also acquired, interspersed throughout the DWI acquisition. DWI were acquired with anterior–posterior phase-encoding. For the correction of susceptibility-related distortions, an additional b = 0 s/mm^2^ image with reversed phase encoding in the posterior–anterior direction was acquired after each DWI acquisition (*82*, *83*). To enhance SNR, each DWI acquisition was repeated twice per participant in different scan sessions. The total DWI acquisition time per session was approximately 34 min.

A single-shot 2D spin-echo echo-planar imaging (SE-EPI) sequence was used [Harvard Medical School, Massachusetts General Hospital [MGH] (*19*, *84*)] in combination with the following imaging parameters: excitation flip angle = 90°, TR = 4600 ms, TE = 75 ms, partial Fourier factor in PE direction = 6/8, in-plane acceleration factor 2 in PE direction using GRAPPA reconstruction, rBW = 1190 Hz/Px, echo spacing = 0.96 ms, multiband factor = 2, acquisition matrix = 210 × 210, field of view (FOV) = 210 × 210 mm^2^, and fat saturation enabled.

#### dMRI analysis

DWI analysis was performed using Matlab version R2022b, MRtrix3 3.0 (*85*), FSL 6.0.3 (*83*, *86*, *87*), and ANTs 2.3.5 (*88*) software packages, as well as the gradunwarp toolbox (*80*) (v1.2.1, https://github.com/Washington-University/gradunwarp) as described below.

#### dMRI preprocessing - distortion correction and quality control

First, the “dwidenoise” and “mrdegibbs” functions implemented in MRtrix3 were used for denoising (*89*, *90*) and correction of Gibbs ringing artifacts (*91*, *92*). Next, the DWI were simultaneously corrected for bulk head motion, eddy current (EC), and tissue susceptibility-induced off-resonance geometric distortions with outlier replacement (*81*, *82*, *93*) using the “dwifslpreproc” function implemented in MRtrix3. The off-resonance field caused by tissue susceptibility variations was estimated using the reversed phase-encoding b = 0 s/mm^2^ image pair with FSL’s “topup” algorithm (*82*, *83*) and subsequently used as input to “eddy_cuda” where a cubic model of the EC field was assumed. Subsequently, the DWI were corrected for spatial distortions due to 3 T Connectom gradient nonlinearity using the gradunwarp toolbox (*80*) and the gradient field specifications provided by the scanner manufacturer. All transforms and warps were combined and applied in a single interpolation step. Finally, the DWI bias field created by the non-uniform coil receive sensitivity was corrected using the “dwibiascorrect” function implemented in MRtrix3, based on the “N4” algorithm implemented in ANTs (*94*).

DWI images were filtered using an automated quality control protocol implemented in FSL *eddy* (*95*). Scans were excluded if they were identified as outliers—defined as having values greater than two standard deviations above or below the group mean—in three or more of the following quality metrics: absolute motion, relative motion, total outlier fraction, percentage of outliers per b-shell, percentage of outliers per phase-encoding direction, contrast-to-noise ratio (CNR) per shell, *eddy* motion parameters (translation, rotation, and eddy-current terms), slice-to-volume motion standard deviation, and susceptibility distortion estimate.

#### dMRI analysis: estimation of diffusion metrics

First, fiber orientation distribution (FOD) maps were estimated using a 3-tissue-compartment constrained spherical deconvolution (CSD) approach (*96*–*100*) necessary for crossing fiber (*101*) and partial volume effect (*102*) decomposition in the superficial white matter and the cortex. Deconvolution kernels were estimated per b-value shell (b = 0, 800, and 1800 s/mm^2^) and per tissue type (anisotropic white matter, slow-decaying isotropic gray matter, fast-decaying isotropic cerebrospinal fluid) using the “dwi2response” function implemented in MRtrix3 using the unsupervised “dhollander” algorithm (*100*). These were then used to estimate the FOD maps by spherical deconvolution (*85*, *97*). Second, diffusion tensor imaging (DTI)-derived parameter maps (*MD*, *RD*, *AD*, FA) were calculated using FSL’s “dtifit” function. Third, *ODI* and *NDI* maps were estimated using the NODDI Matlab toolbox (v1.05, http://www.nitrc.org/projects/noddi_toolbox). To minimize CSF partial volume effects, *NDI* and *ODI* were computed using tissue-weighted means within the V1, V2, and V3 ROIs (*103*).

### Structural MRI

#### sMRI acquisition

For cortex segmentation and image registration, a whole-brain anatomical image was acquired using a T1-weighted MPRAGE sequence (*104*) on the 3 T Connectom scanner with the following parameters: voxel size = 1.0 mm isotropic, TR = 2300 ms, TE = 2.91 ms, FOV = 256 × 240 × 176 mm^3^ (read × phase × partition), rBW = 240 Hz/pixel, and GRAPPA = 2 (primary phase-encoding direction). Four blind participants were unable to fit into the smaller patient bore (approximately 56 cm diameter) of the CONNECTOM scanner (*20*). For three of these participants, T1-weighted images were available from previous studies conducted in Kraków, which we used also in the present analyses. Two of these images were T1-weighted MPRAGE images acquired at the Małopolskie Centrum Biotechnologii using a 3 T Siemens Skyra scanner with a 64-channel head coil (*104*) and the following parameters: voxel size = 0.85 mm isotropic, TR = 1800 ms, TE = 2.37 ms, FOV = 208 × 250 × 250 mm^3^ (read × phase × partition), rBW = 200 Hz/pixel, and GRAPPA = 3 (phase-encoding direction: anterior–posterior). The other image was a T1-weighted MP2RAGE acquired at the Centre for Brain Research using a 3 T Siemens Magnetom Prisma scanner with a 64 channel coil and the following parameters: voxel size = 1.0 mm isotropic, TR = 5000 ms, TE = 2.98 ms, FOV = 256 × 240 × 176 mm^3^ (read × phase × partition), rBW = 240 Hz/pixel, and Grappa = 3 (phase-encoding direction: anterior–posterior). The T1-weighted structural image was derived from the MP2RAGE acquisition using the SPM12 MP2RAGE toolbox (https://github.com/benoitberanger/mp2rage). Background noise and non-brain signal were removed using the toolbox’s built-in masking procedure, yielding a high-contrast, bias-corrected anatomical image.

#### Cortex Segmentation

First, T1-weighted images were corrected for gradient nonlinearities using the gradunwarp toolbox (*80*) (v1.2.1, https://github.com/Washington-University/gradunwarp) and the spherical harmonic coefficients provided by the scanner manufacturer. Second, the resulting images were input to the “recon-all” pipeline in FreeSurfer (*105*) (v7.4.1, http://surfer.nmr.mgh.harvard.edu/) with default parameter settings. A full description of the processing steps can be found elsewhere (*106*). All reconstructed surfaces were visually inspected and manually corrected (see fig. S1 for segmentation quality).

#### Definition of cortical layers and superficial white matter

In a next step, equivolumetric layers between the outer (pial) and the inner (white) surfaces were generated using Surface tools (*107*, *108*) (https://github.com/kwagstyl/surface_tools). The number of computationally defined layers was determined by the image resolution, the assumed thickness of V1 (since this is presumably the thinnest of the three ROIs), and the upsampling factor. In sighted individuals, the average cortical thickness of V1 has been reported to be between 1.9 and 2 mm (*109*). Although there seems to be no consensus in the field regarding the number of computational layers to be extracted, an upsampling factor of four has been recommended (*110*). Thus, a total of 14 intermediate equivolumetric layers were created between the outer and the inner cortical layers for 7 T qMRI (0.5 mm isotropic resolution), 11 layers for 3 T Connectom qMRI (0.8 mm isotropic resolution), and eight layers for 3 T Connectom dMRI (1.0 mm isotropic resolution). These computationally defined cortical layers were then pooled to create superficial, middle, and deep layers for the qMRI data and superficial and deep cortical layers for the dMRI data, given its lower spatial resolution (see table S3 for a summary of the computationally defined cortical layers created for each data type and the resulting superficial, middle, and deep layer specifications). Cortical layers corresponding to the borders between deep, middle and superficial cortical layers were excluded from the analyses, to ensure that the resulting layers are more independent from each other. For the dMRI data, the outer layer was excluded to mitigate contamination of the microstructural measurements by cerebrospinal fluid.

For the definition of the SWM, a surface 0.5 mm below the gray matter/white matter boundary was created. Then, a total of four intermediate equivolume surfaces were created between this surface and the gray matter/white matter boundary using Surface tools (https://github.com/kwagstyl/surface_tools) for the 7 T qMRI data, three surfaces for the 3 T Connectom qMRI data, and two surfaces for the 3 T Connectom dMRI data. The gray matter/white matter boundary surface was excluded from the analyses to reduce partial volume effects from the overlying cortical grey matter.

#### Registration and sampling

All images were registered to the space of the quantitative MRI (qMRI) maps. For registering MPRAGE and MPM images acquired at 3 T, we used the T1w image of the MPRAGE and *MT_sat_* maps (see fig. S1 for registration quality). For registering MPRAGE and MPM images acquired at 7 T, we used the T1w image of the MPRAGE and *R_1_* maps. Using SPM12, brainmasks were generated based on the T1w image of the MPRAGE and the *PD* maps acquired at 3 T and 7 T. To optimize the registration process, the T1w image of the MPRAGE, *MT_sat_*, and *R_1_* maps were corrected for residual bias fields using SPM12, and the brainmasks generated in the previous step were applied. The images were then transformed to a common space via the scanner coordinate system and rigid registration was computed using “FLIRT” (*111*) from FSL (v6.0.7; https://fsl.fmrib.ox.ac.uk/fsl/fslwiki/). The transform between the MPM images acquired at 3 T and diffusion MRI space was computed by a rigid and affine registration in combination with the mutual information (MI) metric in ANTs (ANTs, 2.3.5, http://stnava.github.io/ANTs/) between the *MT_sat_* image and the diffusion MRI-derived FA map. A binary mask was created based on the warped image using FSL’s “BET” tool, to facilitate subsequent non-linear registration. The final transformation was computed with the Symmetric Normalization (SyN) algorithm (*112*) in combination with the cross-correlation (CC) metric. Equivolumetric surfaces were transformed to MPM space using linear interpolation. For data sampling, images were first transformed to MPM space with linear interpolation, then sampled onto the surface mesh using nearest-neighbor interpolation.

### Statistical analyses

Analyses were restricted to V1, V2, and V3, which were defined individually in each participant based on the Benson atlas (*38*).

#### Analysis of morphological features

For each participant’s V1, V2, and V3, we obtained average values of cortical thickness and surface area, curvature, and local gyrification per hemisphere. A linear mixed effects analysis of these parameters was performed using R (v4.2.2) (*113*). As fixed effects we entered group (blind, sighted), ROI (V1, V2, V3), and their interaction terms into the model. Since we did not have hypotheses about the effects of hemisphere on cortical thickness or surface area, this factor was not included in the analysis. As random effects, we included subject-specific intercepts as well as random intercepts and slopes for the effect of ROI within each subject.

If apparent cortical thinning relates to curvature, surface area, or local gyrification, then there should be a significant association between cortical thickness and curvature, surface area, or local gyrification. To be able to test this, we ran three separate models assessing the effect of group and ROI on cortical thickness with curvature, surface area, or local gyrification as a covariate. Moreover, if the reduction in surface area relates to curvature or local gyrification, then there should be a significant association between surface area and curvature or local gyrification. To be able to test this, we ran separate models assessing the effect of group and ROI on surface area with curvature or local gyrification as a covariate. Covariates were mean-centered to facilitate interpretation of fixed effects and to reduce multicollinearity between main effects and interaction terms. P-values for fixed effects were considered significant at *p* < 0.05. Post-hoc comparisons of significant interactions were conducted using approximate z-tests on the estimated marginal means using the emmeans package (*114*). Post-hoc comparisons of slopes were conducted using approximate t-tests on the estimated marginal means of the trends using the emmeans package (*114*). The resulting p-values were corrected for multiple comparisons following the procedure proposed by Holm (*115*).

#### Analysis of microstructural features by intracortical depth

For each participant’s V1, V2, and V3 we obtained average qMRI and dMRI parameters per cortical layer and hemisphere. A generalized linear mixed effects analysis of these parameters (*R_1_*, R_2_^*^, *PD*, *MT_sat_*, *AD*, *MD*, *RD*, *NDI*, *ODI*) was performed using R (v4.2.2) (*113*). As fixed effects we entered group (blind, sighted), ROI (V1, V2, V3), cortical layer (superficial, middle, deep), and their interaction terms into the model. Since we did not have hypotheses about the effects of hemisphere on cortical thickness or surface area, this factor was not included in the analysis. As random effects, we included intercepts for subjects and by subject random intercepts and slopes for the effect of cortical layer. The random effects structure was gradually simplified, until the models reliably converged. Models were first specified for each of these tissue attributes with linear fixed effects for depth, and then with different polynomial interaction terms for depth (for the qMRI measurements). Likelihood ratio tests were used to compare model log-likelihoods, testing whether the addition of the non-linear term yields any robust change in model fit, relative to the simpler alternative model (e.g., linear vs. quadratic) (*116*).

If apparent cortical thinning relates to development in tissue properties, we would expect a significant association between cortical thickness and tissue properties. To test this hypothesis, mean cortical thickness was added as a fixed effect into the same model, as well as its interaction terms with group, ROI, and cortical layer. This allowed us to assess whether cortical qMRI parameters relate to apparent cortical thickness and whether this relationship varies as a function of group, ROI, and cortical layer combination. Cortical thickness was mean-centered to facilitate interpretation of fixed effects and to reduce multicollinearity between main effects and interaction terms. P-values for fixed effects were considered significant at *p* < 0.05. Post-hoc comparisons of significant interactions were conducted using approximate z-tests on the estimated marginal means using the emmeans package (*114*). Post-hoc comparisons of slopes were conducted using approximate t-tests on the estimated marginal means of the trends also using the emmeans package (*114*). The resulting p-values were corrected for multiple comparisons following the procedure proposed by Holm (*115*).

#### Analysis of microstructural features at mid-cortical depth in A1, S1, and M1

To determine whether the effects of reduced myelination were specific to the visual cortex rather than reflecting broader developmental differences, we conducted supplementary analyses of cortical thickness, R_2_^*^ and *MT_sat_* in primary sensory regions (A1, S1, and M1). These ROIs were defined using the Glasser Atlas (*43*). For each participant, we extracted mean cortical thickness, and mean R_2_^*^ and *MT_sat_* values sampled at mid-cortical depth within each ROI. Group differences were assessed using independent-samples, two-sided *t*-tests applied separately to each parameter within each ROI. All statistical tests were performed in R (v4.2.2) (*113*) using the t.test() function. Resulting *p*-values were corrected for multiple comparisons using the false discovery rate (FDR) procedure (Benjamini–Hochberg). ROIs with an FDR-adjusted *p*-value < 0.05 were considered statistically significant.

#### Analysis of microstructural features within the SWM

Average qMRI and dMRI parameters (*R_1_*, R_2_^*^, *PD*, *MT_sat_*, *AD*, *MD*, *RD*, *NDI*, *ODI*) were obtained per hemisphere for each participant’s SWM defined underneath V1, V2, and V3. A generalized linear mixed effects analysis was performed on these parameters using R (v4.2.2) (*113*). As fixed effects we entered group (blind, sighted), ROI (V1, V2, V3), and mean cortical thickness, as well as their interaction terms into the model. Cortical thickness was mean-centered to facilitate interpretation of fixed effects and to reduce multicollinearity between main effects and interaction terms. Since we did not have hypotheses about the effects of hemisphere on the respective measurements, this factor was not included in the analysis. As random effects, we included intercepts for subjects and by subject random intercepts and slopes for the effects of ROI, and their respective interactions. The random effects were gradually simplified, until the models reliably converged. P-values for fixed effects were considered significant at *P* < 0.05. Post-hoc comparisons of significant interactions were conducted using approximate z-tests on the estimated marginal means using the emmeans package (*114*). Post-hoc comparisons of slopes were conducted using approximate t-tests on the estimated marginal means of the trends also using the emmeans package (*114*). The resulting p-values were corrected for multiple comparisons following the procedure proposed by Holm (*115*).

#### Principal component analysis

To identify principal patterns across cortical regions and reduce data dimensionality, we performed a principal component analysis (PCA) on quantitative MRI measures. Prior to PCA, measures were averaged within participants and ROI across cortical depth, based on our univariate analyses showing no significant compartment × group interactions, and to account for differences in the number of computational cortical layers sampled across diffusion and quantitative MRI modalities. PCA was performed across participants and ROIs, such that each participant × ROI combination constituted one observation, and the nine quantitative MRI measures served as input variables. This yielded principal component scores for each ROI and participant. Imaging metrics from multi-parameter mapping (MPM), diffusion-weighted imaging (DWI), and neurite orientation dispersion and density imaging (NODDI) were combined, and only participants with complete data for all nine measures were included. All values were z-scored across participants, and PCA was conducted using the princomp() function in R (v4.2.2) (*113*).

The same linear mixed-effects modeling approach as described above was applied to the first three principal components derived from the PCA analysis, using group, ROI, and mean-centered cortical thickness, as well as their interactions as fixed effects. The random-effects structure included subject-specific intercepts and slopes for ROI and was simplified until the models reliably converged. Post-hoc comparisons and multiple-comparison corrections were performed as described above.

## Supplementary Materials

### This PDF file includes:

Supplementary Text Figs. S1 to S6 Tables S1 to S11 References
