# Supplementary material for: Congenital blindness reduces myelination in human visual cortex
Source: Sci Adv. 2026 Jul 10;12(28):eaec2348. doi: 10.1126/sciadv.aec2348 (PMC13353373; doi:10.1126/sciadv.aec2348)
Supplement: Supplementary file 1 [file sciadv.aec2348_sm.pdf]

Science Advances
AAAS

Supplementary Materials for

Congenital blindness reduces myelination in human visual cortex

Anna-Lena Stroh *et al.*

Corresponding author: Anna-Lena Stroh, annalena.stroh@gmail.com; Marcin Szwed, m.szwed@uj.edu.pl

Sci. Adv. **12**, eaec2348 (2026)
DOI: 10.1126/sciadv.aec2348

This PDF file includes:

Supplementary Text
Figs. S1 to S6
Tables S1 to S11
References

Supplementary Materials

Mechanistic rationale for predicted MRI signatures of pruning and myelination

Pruning and myelination represent distinct, yet temporally overlapping developmental mechanisms that are expected to have opposing effects on microstructure. Conventional MRI lacks the necessary spatial resolution and tissue specificity to disentangle different microstructural mechanisms. However, advanced quantitative (qMRI) techniques now make this feasible as they allow estimation of tissue properties at unprecedented spatial resolutions *in vivo* (*17*, *21*). Using multi-parameter mapping (MPM) it is possible to construct multiple maps of different physical and tissue characteristics (*26*). These include: the longitudinal relaxation rate (R_1), which is sensitive to myelin (*117*), macromolecular content (*118*), iron, and water (*117*); the effective transverse relaxation rate (R_2^*), which is sensitive to iron (*56*, *117*), myelin distribution and fiber orientation (*119*); proton density (*PD*), which is sensitive to the water content (*120*) and decreases as the macromolecular tissue volume (*MTV*) of a voxel increases (*121*) ($MTV = 1 - PD$); and magnetization transfer saturation (MT_{sat}), which is sensitive to myelin and the overall macromolecular content of a voxel (*17*). Like myelination (*10*, *122–124*), R_1, R_2^*, and MT_{sat} parameter values have been shown to decrease from the deeper cortical layers (V/VI) close to the gray–white matter boundary towards the superficial cortical layers (II/I) close to the pial surface (*116*, *125*, *126*). Crucially, MPMs have been shown to be sensitive to neuropathological (*127*, *128*) and age-related changes in myelination (*29*, *129*, *130*). This makes them ideally suited to study the effects of developmental experience on the microstructure of the cortex and allows us to disambiguate developmental hypotheses.

Using dMRI, we can obtain mean diffusivity (*MD*), which depends on the size, density, and structure of the space within tissue through which water diffuses (*10*), radial diffusivity (*RD*), which reflects myelination (*30*), axial diffusivity (*AD*), which has been interpreted to reflect axonal density and/or axonal properties (*131*). We will also obtain more specific microstructural features, such as apparent fiber density (neurite density index; *NDI*) and fiber dispersion (orientation dispersion index; *ODI*) using neurite orientation dispersion and density imaging (NODDI) (*23*). Heavily myelinated white matter bundles typically show lower *ODI* and higher *NDI* (*50*).

Predicted effects of pruning

Synaptic pruning is a key developmental mechanism that refines cortical circuits by eliminating redundant dendritic branches, synapses, and short-range lateral connections, particularly during early visual development (*132*). Efficient pruning is expected to reduce overall cellular and membrane density within a voxel, enlarging the extracellular space, and increasing microstructural coherence. Thus, pruning is *expected to*:

- Reduce macromolecular content, which would lead to reduced R_1, MT_{sat}, and possibly R_2^* values, as these parameters are sensitive to macromolecular and iron content.
- Increase the ease and directionality of water diffusion, resulting in higher *MD* and *AD* (reflecting more directionally coherent fiber architecture).
- Reduce *NDI* and orientation dispersion (*ODI*), consistent with the removal of excess, misaligned processes.

Predicted effects of reduced pruning

If pruning is disrupted or reduced, as has been hypothesized in congenital blindness, the opposite microstructural pattern would be expected. Retained or misaligned dendritic and axonal processes would increase local fiber complexity, thereby reducing coherence of water diffusion and extracellular space, thus leading to more restricted and less coherent diffusion (reduced *MD* and *AD*) and to increased neurite density and orientation dispersion along with reduced *PD*. The increase in unmyelinated cellular and membrane content would increase the overall macromolecular content within a voxel, leading to increased R_1, MT_{sat}, and R_2^*.

Predicted effects of myelination

During myelination, the development of lipid-rich sheaths is expected to increase the macromolecular content (*10*, *29*) and reduce extracellular water (*133*). This process starts later and over a more extended timescale than pruning and continues through adolescence and early adulthood (*9*). The increase in myelin and macromolecular content is expected to:

- Increase R_1 and R_2^*, as both parameters are sensitive to myelin, iron, and overall macromolecular content.
- Increase MT_{sat}, which is sensitive to myelin and overall macromolecular content.
- Reduce *PD*, *MD*, and *RD* due to decreased extracellular water and restricted diffusion perpendicular to fibers.
- Increase *NDI* reflecting greater axonal packing density.

Empirical support for these relationships comes from developmental MRI studies reporting age-related increases in R_1, MT_{sat}, and *NDI*, together with decreases in *MD* and *RD* across the cortex during infancy and childhood (*10*, *29*). However, these associations are indirect and reflect model-based interpretations. Future developmental studies using histology are needed to confirm the expected relationships between changes in MRI parameters and underlying cellular developmental mechanisms.

Predicted effects of reduced myelination

If myelination is reduced in congenitally blind individuals, the inverse pattern would be expected:

- reduced R_1, R_2^*, and MT_{sat} values (reflecting reduced macromolecular and myelin content),
- increased *PD*, *MD* and *RD* (reflecting less restricted diffusion),
- and reduced *NDI* (reflecting reduced axonal packing).

These differences would be most pronounced in deeper cortical layers and superficial white matter, where myelination is normally highest.

These predictions are therefore inverse to those associated with reduced pruning and serve as a conceptual framework to distinguish whether observed MRI differences primarily reflect altered synaptic pruning or myelination. We emphasize that these relationships are model-based and inferential, not based on histological experiments. Pruning and myelination likely act together during normal development, and both may be altered in blindness. The net effect on MRI parameters would thus depend on the relative magnitude of these competing influences.

Predicted combined effects of reduced pruning and reduced myelination

Reduced pruning could increase the density of synapses, dendrites, and unmyelinated axons, increasing macromolecular content and restricting water diffusion; whereas reduced myelination could reduce myelin-associated macromolecules and increase extracellular water content. The net effect on MRI parameters would therefore vary depending on which process dominates:

- If reduced pruning dominates, we expect higher MT_{sat}, R_1, R_2^*, and *NDI*, along with lower *PD*, *MD,* and *AD* in blind compared to sighted individuals.
- If reduced myelination dominates, we expect lower MT_{sat}, R_1, R_2^*, and *NDI*, along with higher *PD*, *MD* and *RD* in blind compared to sighted individuals. *AD* is not expected to change.
- Concurrent effects of reduced pruning and myelination should lead to increased *RD* and *ODI*, independent of which process dominates.
- If both effects are comparable, their opposing influences could offset one another, resulting in no significant difference between groups. The net effect on MRI parameters would thus depend on the relative magnitude of these competing influences.

Note that as part of the review process, we have adjusted the hypotheses for *MD, AD,* and *NDI* in the case of concurrent effects, to avoid overinterpretation of our data. It should be mentioned that we included the assessment of g-ratio estimates in the preregistration, since it will be the target of future analyses.

Differences in Morphology

Congenitally blind individuals have reduced surface area, increased curvature, and decreased gyrification in the early visual cortex. Consistent with our hypotheses, the group of blind individuals had reduced surface area in the early visual cortex. The effect of group varied by ROI (fig. S4A, group x ROI interaction: $F_{(2, 231.00)} = 16.25$, $p < 0.001$), with the largest reductions of surface area in V1 (group differences V1: -615 mm^2, V2: -325 mm^2, V3: -246 mm^2, all *ps* < 0.05, table S11). The analysis of mean cortical curvature revealed that blind individuals had increased cortical curvature within early visual cortices compared to sighted controls (fig. S4B, main effect of group: $F_{(1, 45.00)} = 46.28$, $p < 0.001$; group difference: 0.015 mm^{-1} ± 0.002 mm^{-1}, $df = 45$, *t-ratio* = 6.803, $p < 0.001$). Moreover, blind individuals showed reduced gyrification in early visual cortices compared to sighted controls (fig. S4C, main effect of group: $F_{(1, 42.00)} = 14.02$, $p < 0.001$; group difference: -0.159 ± 0.042, $df = 45$, *t-ratio* = -3.744, $p < 0.001$).

Surface area scales with local gyrification. If the reduction in surface area relates to curvature or local gyrification, then there should be a significant association between surface area and curvature or local gyrification. To be able to test this, we ran separate models assessing the effect of group and ROI on surface area with curvature or local gyrification as a covariate. We observed a significant group by ROI by curvature interaction ($F_{(2, 145.99)} = 14.19$, $p < 0.001$), suggesting that the effect of curvature differed as a function of group and ROI combination. In V1 of sighted controls, there was a positive association between surface area and curvature (slope in sighted controls: 125.50 cm^2/mm^{-1}, *CI* = [41.0, 210.0] cm^2/mm^{-1}, slope in blind individuals: -9.93 cm^2/mm^{-1}, CI = [-85.8, 66.0] cm^2/mm^{-1}). Thus, the larger the surface area of V1 in sighted controls, the larger the mean curvature, suggesting that sighted controls with larger V1s had more sulcal area. In addition, we observed a positive association between surface area and local gyrification (fig. S4D, $F_{(1, 263.9)} = 70.91$, $p < 0.001$; slope: 10.56 cm^2, CI = [8.07, 13.10] cm^2). That is, individuals with larger early visual cortices also had more gyrified early visual cortices. This is in line with the finding that the degree of cortical folding scales uniformly across species, individuals, and within individual cortices with surface area (*134*).

Associations between Microstructure of the Gray Matter and Cortical Thickness

Lower myelin content of the gray matter is associated with apparent thicker early visual cortex in blind individuals. In addition, the analysis of the 3T data revealed a negative association between MT_{sat} and cortical thickness in blind individuals (fig. S5A, group × cortical thickness interaction: $F_{(1, 620.09)} = 6.78$, $p = 0.009$, slope in blind individuals: -0.0587 p.u./mm, CI = [-0.1069, -0.0104] p.u./mm, sighted controls: 0.0333 p.u./mm, CI = [-0.0136, 0.0802] p.u./mm). These results suggest that blind individuals with thicker early visual cortices had lower myelination of this region. Moreover, in the middle layer of blind individuals, we observed a positive association between *PD* and cortical thickness (fig. S5B, group × layer × cortical thickness interaction: $F_{(2, 585.63)} = 3.54$, $p = 0.03$, slope in blind individuals: 1.753 p.u./mm, CI = [0.165, 3.34] p.u./mm, all other slopes not significantly different from 0). Thus, blind individuals with apparent thicker early visual cortices had higher *PD* in the middle layer of the early visual cortex.

The analysis of the 7T data revealed a negative association between *PD* and cortical thickness ($F_{(1, 240.25)} = 13.86$, $p < 0.001$), slope: -2.6 p.u./mm, CI = [-5.19, -0.0123] p.u./mm).

Lower diffusivity of the gray matter is associated with apparent thicker early visual cortex. In blind individuals, *AD* and cortical thickness were negatively associated in V1 and V2 (fig. S5C, group × ROI × cortical thickness interaction: $F_{(2, 368.86)} = 3.33$, $p = 0.037$, slope in V1 of blind

individuals: -0.09271 mm^2s^{-1}/mm, CI = [-0.1291, -0.05633] mm^2s^{-1}/mm, sighted controls: 0.00595 mm^2s^{-1}/mm, CI = [-0.0273, 0.03923] mm^2s^{-1}/mm, in V2 of blind individuals: -0.04328 mm^2s^{-1}/mm, CI = [-0.0774, -0.00918] mm^2s^{-1}/mm, in sighted controls: 0.0077 mm^2s^{-1}/mm, CI = [-0.0260, 0.04144] mm^2s^{-1}/mm, slopes in V3 not different from 0). Moreover, in blind individuals, *MD* of the early visual cortex and cortical thickness were negatively associated (fig. S5D, group × cortical thickness interaction: $F_{(1, 403.72)} = 5.06$, $p = 0.025$, slope in blind individuals: -0.03681 mm^2s^{-1}/mm, CI = [-0.0576, -0.016] mm^2s^{-1}/mm, sighted controls: -0.00192 mm^2s^{-1}/mm, CI = [-0.0245, 0.0206] mm^2s^{-1}/mm. In addition, we observed a negative association between *RD* and cortical thickness in V1 (fig. S5E, ROI × cortical thickness interaction: $F_{(2, 372.05)} = 10.81$, $p < 0.001$, slope in V1: -0.04971 mm^2s^{-1}/mm, CI = [-0.0706, -0.0288] mm^2s^{-1}/mm).

More densely packed gray matter is associated with apparent thicker early visual cortex. There was a positive association between cortical thickness and neurite density index (*NDI*) in the early visual cortex (fig. S5F, main effect of cortical thickness: $F_{(1, 395.57)} = 4.43$, $p = 0.036$, slope: 0.0177 mm^{-1}, CI = [0.00112, 0.0342] mm^{-1}). Moreover, sighted controls but not blind individuals showed a negative association between cortical thickness and orientation dispersion index (*ODI*) in the deep layer of the early visual cortex (fig. S5G, group × layer × cortical thickness interaction: $F_{(1, 33.92)} = 8.16$, $p = 0.005$, slope in deep layer in sighted controls: -0.06956 mm^{-1}, CI = [-0.0947, -0.04445] mm, group of blind individuals: -0.00493 mm^{-1}, CI = [-0.0285, 0.01862] mm^{-1}). Taken together, these results suggest that individuals with more densely packed tissue (higher *NDI* and lower *ODI*) in the early visual cortex have an apparent thicker cortex.

<u>Associations between Microstructure of the Superficial White Matter and Cortical Thickness</u>

The analysis of the R_2^* 3T data revealed a group by ROI by thickness interaction (fig. S6A, $F_{(2, 196.25)} = 7.27$, $p < 0.001$), suggesting that the association between R_2^* and cortical thickness differed as a function of group and ROI combination. There was a negative association between R_2^* in the SWM of V3 and cortical thickness in blind individuals, that is, blind individuals with thicker V3s had lower R_2^* in the adjoining white matter (slope in group of blind individuals: -2.009 s^{-1}/mm, CI = [-3.590, -0.425] s^{-1}/mm, slope sighted controls: -0.953 s^{-1}/mm, CI = [-2.400, 0.498] s^{-1}/mm). In V1, we observed the opposite trend, that is, blind individuals with thicker V1s had higher R_2^* in the adjoining white matter (slope in blind individuals: 2.886 s^{-1}/mm, CI = [1.480, 4.298] s^{-1}/mm, sighted controls: -0.229 s^{-1}/mm, CI = [-1.520, 1.059] s^{-1}/mm). Sighted controls, but not blind individuals showed a positive association between MT_{sat} and cortical thickness (fig. S6B, group × cortical thickness interaction: $F_{(1, 255.94)} = 4.46$, $p = 0.036$), slope sighted controls: 0.14041 p.u./mm, CI = [0.0466, 0.2342] p.u./mm, group of blind individuals: 0.00342 p.u./mm, CI = [-0.0843, 0.0912] p.u./mm). That is, sighted controls with thicker early visual cortices had higher MT_{sat} in the SWM of the early visual cortex. Moreover, individuals with thicker early visual cortices had lower *PD* in the SWM of this region (fig. S6D, main effect of thickness: $F_{(1, 233.90)} = 15.22$, $p < 0.001$, slope: -1.98 p.u./mm, CI = [-2.98, -0.97] p.u./mm).

The analysis of the 7T data revealed similar patterns. We observed a negative association between R_2^* and cortical thickness in the SWM of the early visual cortex ($F_{(1, 134.96)} = 7.46$, $p = 0.007$, slope: -2.51 s^{-1}/mm, CI = [-4.44, -0.589] s^{-1}/mm). In addition, there was a negative association between *PD* of the SWM and cortical thickness in sighted controls (group × cortical thickness interaction: $F_{(1, 163.45)} = 5.91$, $p = 0.016$), slope in blind individuals: -1.37 p.u./mm, CI = [-4.09, 1.35] p.u./mm, sighted controls: -5.88 p.u./mm, CI = [-8.41, -3.34] p.u./mm).

Lower diffusivity of the superficial white matter is associated with apparent thicker cortex. In blind individuals, *AD* of the SWM and cortical thickness were negatively associated, that is,

blind individuals with thicker early visual cortices had lower *AD* (fig. S6E, group × cortical thickness interaction: $F_{(1, 216.09)} = 11.46$, $p < 0.001$, slope in blind individuals: -5.9×10^{-6} mm^2s^{-1}/mm, CI = [-0.0976, -0.0205] mm^2s^{-1}/mm, sighted controls: 3.5×10^{-6} mm^2s^{-1}/mm, CI = [-0.0048, 0.0747] mm^2s^{-1}/mm). *MD* of the SWM underneath the early visual cortex and cortical thickness were negatively associated in blind individuals (fig. S6F, group × cortical thickness interaction: $F_{(1, 221.10)} = 6.81$, $p = 0.01$, slope in blind individuals: -4.764×10^{-6} mm^2s^{-1}/mm, CI = [-0.0726, -0.0227] mm^2s^{-1}/mm, sighted controls: 0.0138×10^{-6} mm^2s^{-1}/mm, CI = [-0.0264, 0.0266] mm^2s^{-1}/mm). In addition, we observed a negative association between *RD* and cortical thickness in V1 (fig. S6G, ROI × cortical thickness interaction: $F_{(2, 186.71)} = 7.48$, $p < 0.001$, slope in V1: -6.147×10^{-6} mm^2s^{-1}/mm, CI = [-0.0867, -0.0362] mm^2s^{-1}/mm, all other slopes not significantly different from 0).

More densely packed superficial white matter is associated with apparent thicker cortex. There was a positive association between cortical thickness and *NDI* in the SWM of the early visual cortex (fig. S6H, $F_{(1, 214.16)} = 10.87$, $p = 0.001$, slope: 0.050 mm^{-1}, CI = [0.0199, 0.0797] mm^{-1}). Moreover, sighted controls showed a negative association between cortical thickness and *ODI* in V1 and V2 (fig. S6I, group × ROI × cortical thickness interaction: $F_{(2, 187.53)} = 3.14$, $p = 0.045$, slope sighted controls: -0.1266 mm^{-1}, CI = [-0.1702, -0.0831] mm^{-1}, group of blind individuals: -0.0295 mm^{-1}, CI = [-0.0776, 0.01856] mm^{-1}) and V2 (slope sighted controls: -0.0531 mm^{-1}, CI = [-0.0971, -0.00903] mm^{-1}, group of blind individuals: 0.0127 mm^{-1}, CI = [-0.0324, 0.05775] mm^{-1}).

Supplementary Figures

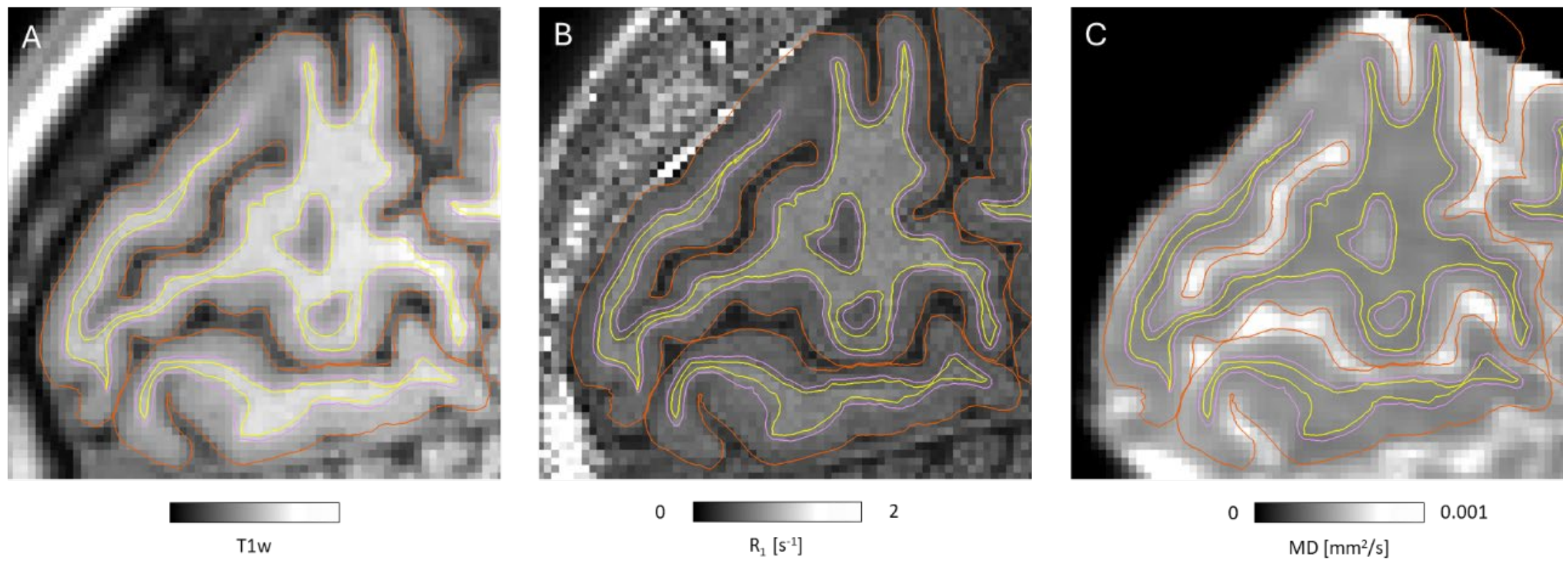

fig. S1 Illustration of the segmentation and registration quality of 3T data. (**A**) Posterior part of the MPRAGE image in sagittal orientation of one representative participant (occipital pole on the left side of the image), which was used for segmentation of the cerebral cortex. Overlaid contour lines show the reconstructed pial boundary surface (orange), the white matter/gray matter boundary surface (pink), and a surface that was created 0.5 mm below the white matter/gray matter boundary surface in the superficial white matter (yellow). (**B**) R_1 map computed from the multi-parameter mapping (MPM) acquisition. (**C**) *MD* map derived from DTI. Overlaid contour lines are identical to (**A**) to visualize the segmentation and registration quality.

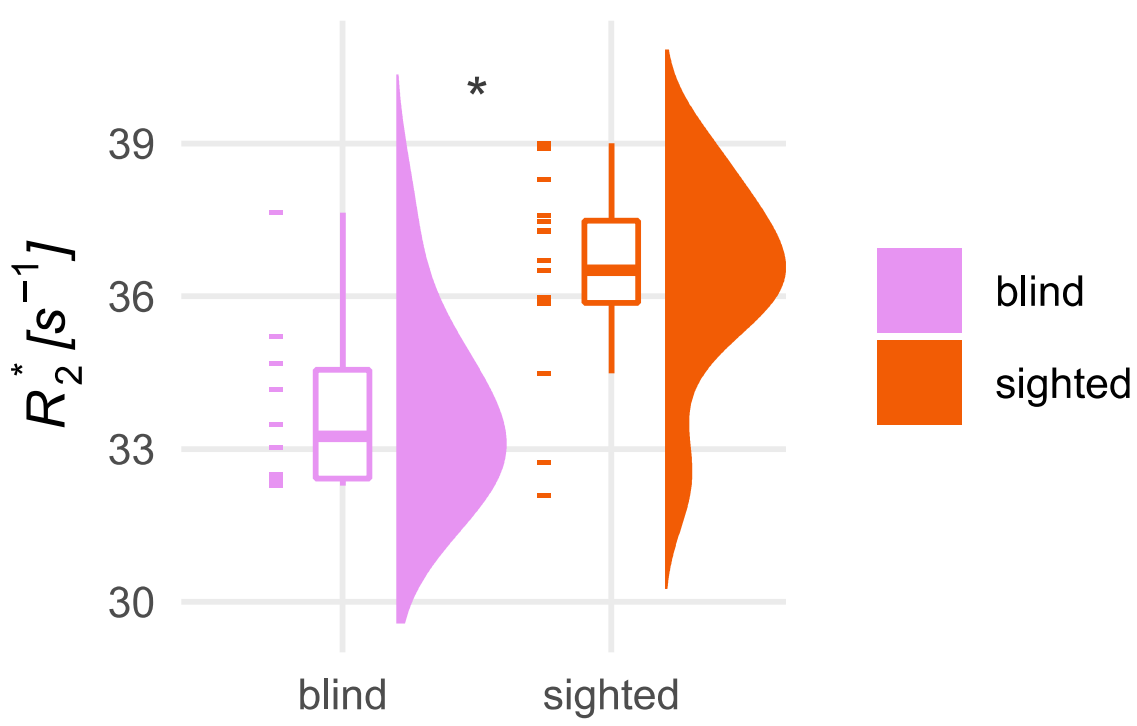

fig. S2 Lower *R2 in the early visual cortex of congenitally blind individuals at 7T.** The distributions of R_2^* parameter values are shown on raincloud and boxplots. Boxplots represent minimum, quartile 1, median, quartile 3, and maximum. Individual data points represent the mean parameter per participant. R_2^* = effective transverse relaxation rate.

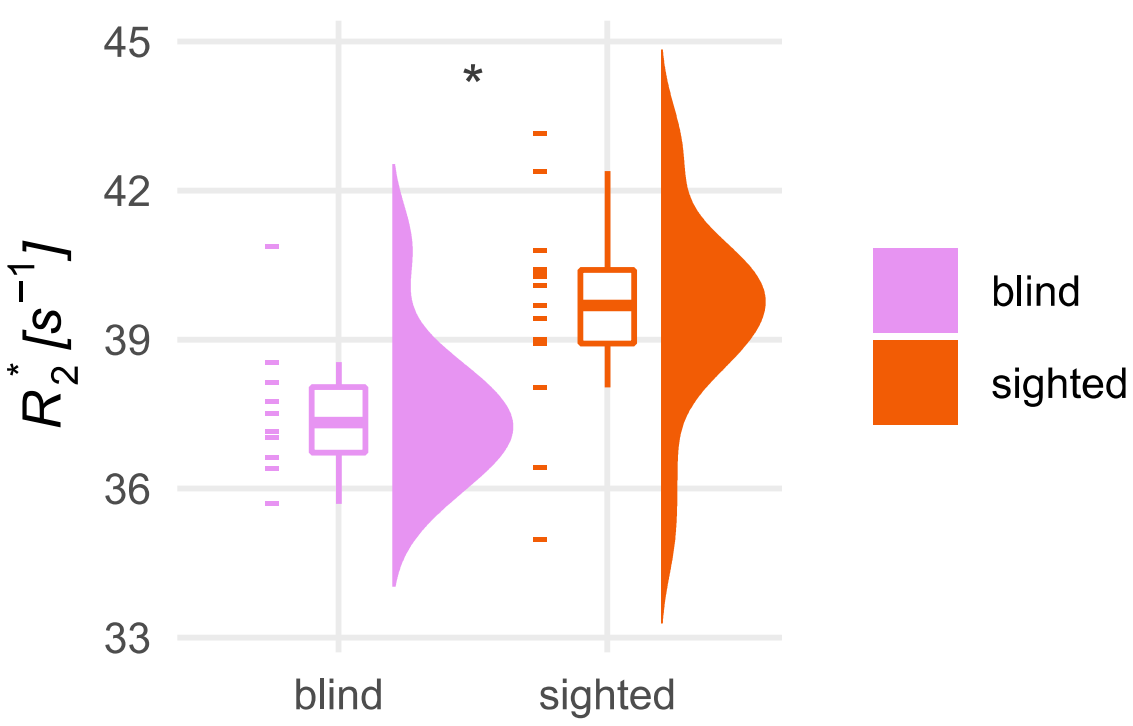

fig. S3 Lower *R2 in the superficial white matter underneath the early visual cortex of congenitally blind individuals at 7T.** The distributions of R_2^* parameter values are shown on raincloud and boxplots. Boxplots represent minimum, quartile 1, median, quartile 3, and maximum. Individual data points represent the mean parameter per participant. R_2^* = effective transverse relaxation rate.

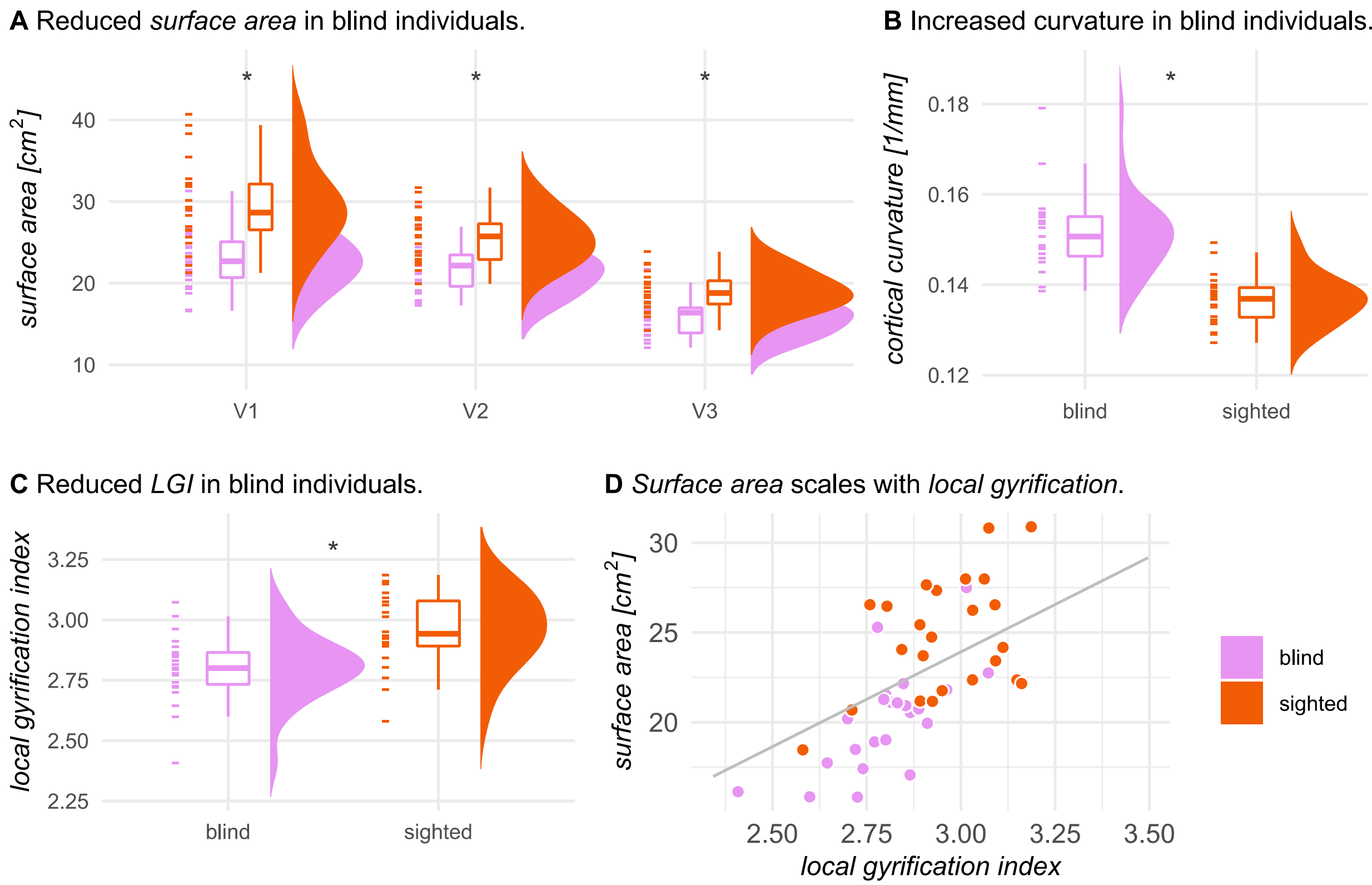

fig. S4 Blind individuals have reduced surface area and local gyrification, along with increased cortical curvature. Raincloud and boxplots show the distribution of cortical thickness across cortical regions (V1, V2, V3) and group (blind, sighted). Box plots represent minimum, quartile 1, median, quartile 3, and maximum. (**A**) Surface area was reduced in blind individuals, with the biggest difference in V1. Individual data points represent the mean surface area per participant and region. (**B**) Cortical curvature was increased in blind individuals. Individual data points represent the mean cortical curvature per participant. (**C**) Local gyrification index was reduced in blind individuals. Individual data points represent the mean local gyrification per participant. (**D**) Positive association between surface area and local gyrification. Individual data points represent the mean surface area plotted against the mean local gyrification index per participant.

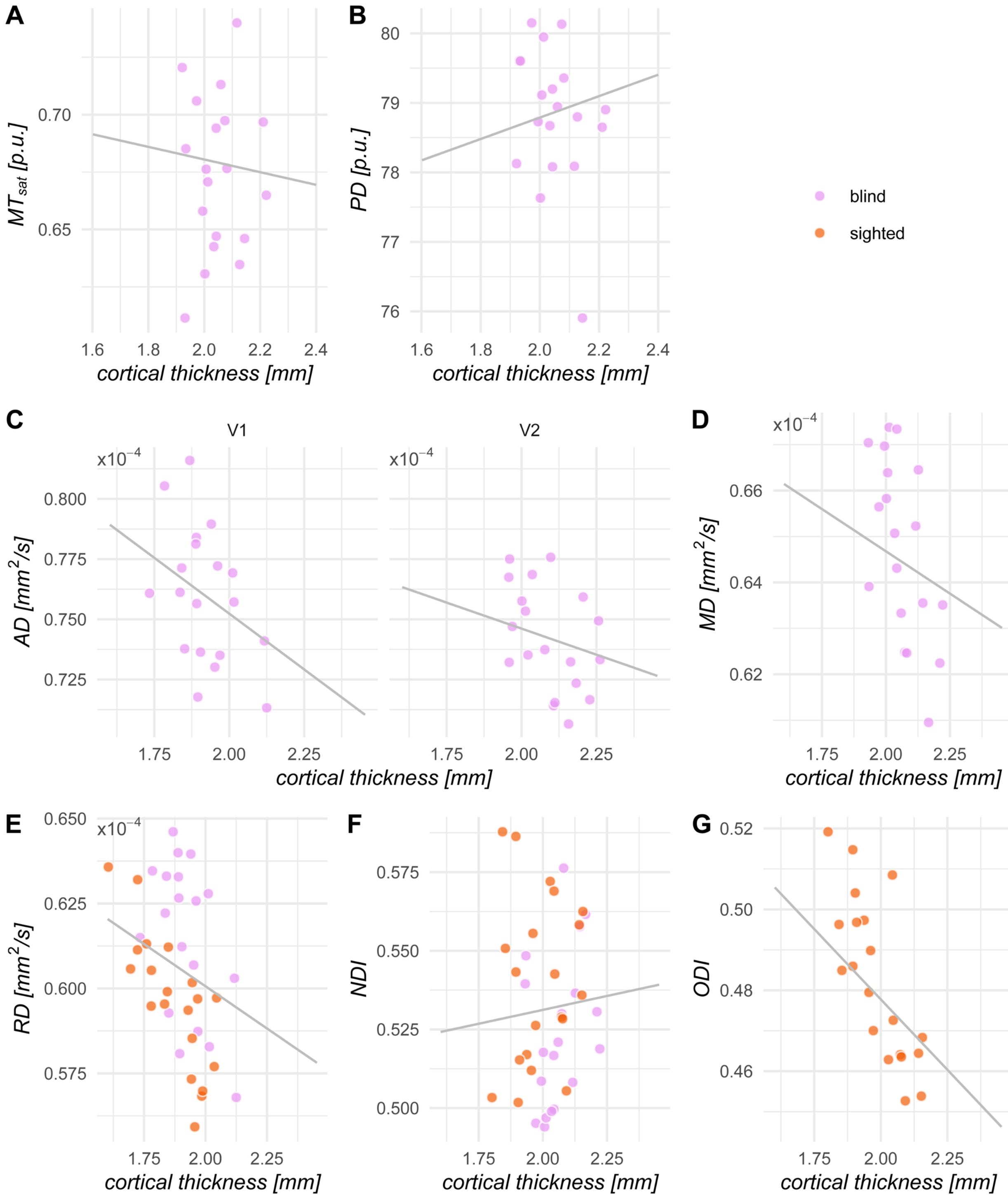

fig. S5 Associations between gray matter microstructure and cortical thickness in the early visual cortex. (**A**) Negative association between MT_{sat} and cortical thickness in the early visual cortex of blind individuals. (**B**) Positive association between *PD* and cortical thickness in middle layer of the early visual cortex in blind individuals. (**C**) Negative association between *AD* and cortical thickness in V1 and V2 of blind individuals. (**D**) Negative association between *MD* and cortical thickness in blind individuals. (**E**) Negative association of *RD* and cortical thickness in V1. (**F**) Positive association between *NDI* and cortical thickness. (**G**) Negative association between

ODI and cortical thickness in the deep layer of the early visual cortex. Note that cortical thickness is shown in its original (non-centered) form to aid comparability with previous studies. However, all statistical analyses were conducted using mean-centered cortical thickness values. *AD* = axial diffusivity, *MD* = mean diffusivity, MT_{sat} = magnetization transfer saturation, *NDI* = neurite density index, *ODI* = orientation dispersion index, *PD* = proton density, *RD* = radial diffusivity.

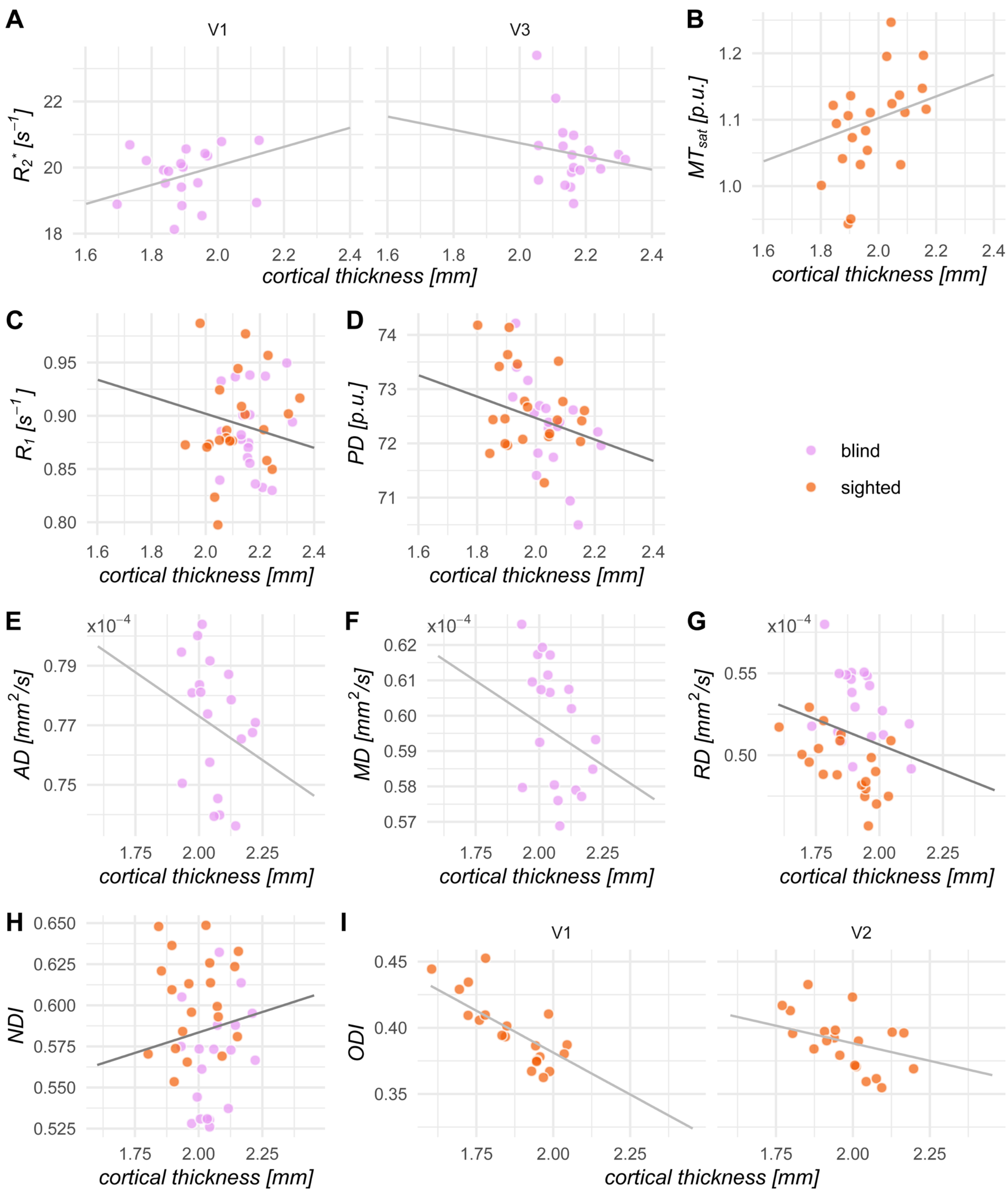

fig. S6 Associations between microstructure in the superficial white matter and cortical thickness in the early visual cortex. (**A**) Positive association between R_2^* and cortical thickness in V1 and negative association between R_2^* and cortical thickness in V3 of blind individuals. (**B**) Positive association between MT_{sat} and cortical thickness in sighted controls. (**C**) Negative association between R_1 and cortical thickness. (**D**) Negative association between *PD* and cortical

thickness. (**E-F**) Negative association between cortical thickness and *AD* (**E**) and *MD* (**F**) in blind individuals. (**G**) Negative association between *RD* and cortical thickness. (**H**) Positive association between *NDI* and cortical thickness. (**I**) Negative association between *ODI* and cortical thickness in V1 and V2 of sighted controls. Note that cortical thickness is shown in its original (non-centered) form to aid comparability with previous studies. However, all statistical analyses were conducted using mean-centered cortical thickness values. *AD* = axial diffusivity, *MD* = mean diffusivity, MT_{sat} = magnetization transfer saturation, *NDI* = neurite density index, *ODI* = orientation dispersion index, *PD* = proton density, R_1 = longitudinal relaxation rate, R_2^* = effective transverse relaxation rate, *RD* = radial diffusivity.

Supplementary tables

table S1 Number of participants per analysis.

analysis	n blind	n sighted
**gross morphology** (cortical thickness, surface area, curvature, and local gyrification)	23	24
**MPMs at 3T**	19	22
**DTI-derived metrics**	19	20
**MPMs at 7T**		
- R_2^* & *PD*	10	17
- R_1	13	17

table S2 Reasons for missing and/or excluded data per analysis.

field strength	analysis	n blind	n sighted
**3T - qMRI**	physical constraints precluding comfortable positioning in the scanner	4	0
	head motion	1	1
	technical error	0	1
**3 T - dMRI**	physical constraints precluding comfortable positioning in the scanner	4	0
	did not pass qc	1	3
	no MPM scans available for registration	0	1
**7T - qMRI**	head motion - *R2** & *PD*	12	7
	head motion - R_1	8	7
	technical error	1	0
	scan not completed due to participant discomfort	1	0

table S3 Summary of the computationally defined cortical layers created using an equivolume model for each data type and the resulting superficial, middle, and deep cortical layer definitions.

layer	qMRI data at 0.5 mm resolution	qMRI data at 0.8 mm resolution	DWI data at 1 mm resolution
**total**	**14 layers**	**11 layers**	**8 layers**
superficial	layers 1:4	layers 1:3	layers 2:4
middle	layers 6:9	layers 5:7	
deep	layers 11:14	layers 9:11	layers 6:8

table S4 Results of post hoc tests assessing the effect of group (blind vs sighted) on R_2^* of the gray matter within each ROI.

ROI	*estimate*	*SE*	*df*	*t-ratio*	*p-value*
V1	-1.65 s^{-1}	0.336 s^{-1}	58.982	-4.901	< 0.001
V2	-1.21 s^{-1}	0.334 s^{-1}	44.546	-3.634	0.001
V3	-1.06 s^{-1}	0.368 s^{-1}	59.437	-2.874	0.006

table S5 Results of post hoc tests assessing the effect of group (blind vs sighted) on MT_{sat} of the gray matter within each ROI.

ROI	*estimate*	*SE*	*df*	*t-ratio*	*p-value*
V1	-0.072 p.u.	0.015 p.u.	67.469	-4.845	< 0.001
V2	-0.036 p.u.	0.014 p.u.	51.879	-2.615	0.023
V3	-0.031 p.u.	0.015 p.u.	77.994	-1.993	0.05

table S6 Results of post hoc tests assessing the effect of group (blind vs sighted) on *AD* of the gray matter within each ROI.

ROI	*estimate*	*SE*	*df*	*t-ratio*	*p-value*
V1	0.527 x 10^{-6} mm^2/s	0.669 x 10^{-6} mm^2/s	73.259	0.787	0.434
V2	1.959 x 10^{-6} mm^2/s	0.602 x 10^{-6} mm^2/s	50.728	3.252	0.005
V3	2.161 x 10^{-6} mm^2/s	0.664 x 10^{-6} mm^2/s	72.470	3.252	0.005

table S7 Results of post hoc tests assessing the effect of group (blind vs sighted) on *AD* of the superficial white matter within each ROI.

ROI	*estimate*	*SE*	*df*	*t-ratio*	*p-value*
V1	-0.737 x 10^{-6} mm^2/s	0.814 x 10^{-6} mm^2/s	113.192	-0.905	0.367
V2	2.599 x 10^{-6} mm^2/s	0.676 x 10^{-6} mm^2/s	71.929	3.844	0.001
V3	2.684 x 10^{-6} mm^2/s	0.809 x 10^{-6} mm^2/s	117.091	3.318	0.002

table S8 Results of post hoc tests assessing the effect of group (blind vs sighted) on the neurite density index (*NDI*) of the superficial white matter within each ROI.

ROI	*estimate*	*SE*	*df*	*t-ratio*	*p-value*
V1	-0.025	0.011	68.706	-2.19	0.032
V2	-0.045	0.01	49.193	-4.347	< 0.001
V3	-0.044	0.011	67.870	-3.911	< 0.001

table S9 Results of post hoc tests assessing the effect of group (blind vs sighted) on *PC1* within the SWM underneath each ROI.

ROI	*estimate*	*SE*	*df*	*t-ratio*	*p-value*
V1	-1.366	0.491	58.229	-2.783	0.007
V2	-2.145	0.455	44.631	-4.713	< 0.001
V3	-2.136	0.493	59.814	-4.329	< 0.001

table S10 Results of post hoc tests assessing the effect of group (blind vs sighted) on cortical thickness within each ROI.

ROI	*estimate*	*SE*	*df*	*t-ratio*	*p-value*
V1	0.055 mm	0.033 mm	62.458	1.665	0.101
V2	0.126 mm	0.033 mm	62.458	3.849	< 0.001
V3	0.058 mm	0.033 mm	62.458	1.782	0.08

table S11 Results of post hoc tests assessing the effect of group (blind vs sighted) on surface area within each ROI.

ROI	*estimate*	*SE*	*df*	*t-ratio*	*p-value*
V1	-6.15 cm^2	0.826 cm^2	72.359	-7.443	< 0.001
V2	-3.25 cm^2	0.826 cm^2	72.359	-3.935	< 0.001
V3	-2.46 cm^2	0.826 cm^2	72.359	-2.98	0.004

REFERENCES

1. J. H. Gilmore, R. C. Knickmeyer, W. Gao, Imaging structural and functional brain development in early childhood. *Nat. Rev. Neurosci.* **19**, 123–137 (2018).

2. J. C. Silbereis, S. Pochareddy, Y. Zhu, M. Li, N. Sestan, The cellular and molecular landscapes of the developing human central nervous system. *Neuron* **89**, 248–268 (2016).

3. I. Bystron, P. Rakic, Z. Molnár, C. Blakemore, The first neurons of the human cerebral cortex. *Nat. Neurosci.* **9**, 880–886 (2006).

4. B. H. Choi, L. W. Lapham, Radial glia in the human fetal cerebrum: A combined golgi, immunofluorescent and electron microscopic study. *Brain Res.* **148**, 295–311 (1978).

5. L. C. deAzevedo, C. Fallet, V. Moura-Neto, C. Daumas-Duport, C. Hedrin-Pereira, R. Lent, Cortical radial glial cells in human fetuses: Depth-correlated transformation into astrocytes. *J. Neurobiol.* **55**, 288–298 (2003).

6. M. S. Y. Yeung, S. Zdunek, O. Bergmann, S. Bernard, M. Salehpour, K. Alkass, S. Perl, J. Tisdale, G. Possnert, L. Brundin, H. Druid, J. Frisén, Dynamics of oligodendrocyte generation and myelination in the human brain. *Cell* **159**, 766–774 (2014).

7. P. R. Huttenlocher, C. De Courten, L. J. Garey, H. Van Der Loos, Synaptogenesis in human visual cortex — evidence for synapse elimination during normal development. *Neurosci. Lett.* **33**, 247–252 (1982).

8. P. R. Huttenlocher, A. S. Dabholkar, Regional differences in synaptogenesis in human cerebral cortex. *J. Comp. Neurol.* **387**, 167–178 (1997).

9. I. Kostović, G. Sedmak, M. Judaš, Neural histology and neurogenesis of the human fetal and infant brain. *Neuroimage* **188**, 743–773 (2019).

10. V. S. Natu, J. Gomez, M. Barnett, B. Jeska, E. Kirilina, C. Jaeger, Z. Zhen, S. Cox, K. S. Weiner, N. Weiskopf, K. Grill-Spector, Apparent thinning of human visual cortex during

childhood is associated with myelination. *Proc. Natl. Acad. Sci. U.S.A.* **116**, 20750–20759 (2019).

11. D. J. Miller, T. Duka, C. D. Stimpson, S. J. Schapiro, W. B. Baze, M. J. McArthur, A. J. Fobbs, A. M. M. Sousa, N. Šestan, D. E. Wildman, L. Lipovich, C. W. Kuzawa, P. R. Hof, C. C. Sherwood, Prolonged myelination in human neocortical evolution. *Proc. Natl. Acad. Sci. U.S.A.* **109**, 16480–16485 (2012).

12. O. De Faria, H. Pivonkova, B. Varga, S. Timmler, K. A. Evans, R. T. Káradóttir, Periods of synchronized myelin changes shape brain function and plasticity. *Nat. Neurosci.* **24**, 1508–1521 (2021).

13. J. Jiang, W. Zhu, F. Shi, Y. Liu, J. Li, W. Qin, K. Li, C. Yu, T. Jiang, Thick visual cortex in the early blind. *J. Neurosci.* **29**, 2205–2211 (2009).

14. H.-J. Park, J. D. Lee, E. Y. Kim, B. Park, M.-K. Oh, S. Lee, J.-J. Kim, Morphological alterations in the congenital blind based on the analysis of cortical thickness and surface area. *Neuroimage* **47**, 98–106 (2009).

15. A. Inuggi, A. Pichiecchio, B. Ciacchini, S. Signorini, F. Morelli, M. Gori, Multisystemic increment of cortical thickness in congenital blind children. *Cereb. Cortex Commun.* **1**, tgaa071 (2020).

16. A.-L. Stroh, D. Radziun, M. Korczyk, L. Crucianelli, H. H. Ehrsson, M. Szwed, Blind individuals' enhanced ability to sense their own heartbeat is related to the thickness of their occipital cortex. *Cereb. Cortex* **34**, bhae324 (2024).

17. N. Weiskopf, L. J. Edwards, G. Helms, S. Mohammadi, E. Kirilina, Quantitative magnetic resonance imaging of brain anatomy and in vivo histology. *Nat. Rev. Phys.* **3**, 570–588 (2021).

18. F. Movahedian Attar, E. Kirilina, D. Haenelt, K. J. Pine, R. Trampel, L. J. Edwards, N. Weiskopf, Mapping short association fibers in the early cortical visual processing stream using in vivo diffusion tractography. *Cereb. Cortex* **30**, 4496–4514 (2020).

19. K. Setsompop, R. Kimmlingen, E. Eberlein, T. Witzel, J. Cohen-Adad, J. A. McNab, B. Keil, M. D. Tisdall, P. Hoecht, P. Dietz, S. F. Cauley, V. Tountcheva, V. Matschl, V. H. Lenz, K. Heberlein, A. Potthast, H. Thein, J. Van Horn, A. Toga, F. Schmitt, D. Lehne, B. R. Rosen, V. Wedeen, L. L. Wald, Pushing the limits of in vivo diffusion MRI for the Human Connectome Project. *Neuroimage* **80**, 220–233 (2013).

20. Q. Fan, C. Eichner, M. Afzali, L. Mueller, C. M. W. Tax, M. Davids, M. Mahmutovic, B. Keil, B. Bilgic, K. Setsompop, H.-H. Lee, Q. Tian, C. Maffei, G. Ramos-Llordén, A. Nummenmaa, T. Witzel, A. Yendiki, Y.-Q. Song, C.-C. Huang, C.-P. Lin, N. Weiskopf, A. Anwander, D. K. Jones, B. R. Rosen, L. L. Wald, S. Y. Huang, Mapping the human connectome using diffusion MRI at 300 mT/m gradient strength: Methodological advances and scientific impact. *Neuroimage* **254**, 118958 (2022).

21. N. Weiskopf, S. Mohammadi, A. Lutti, M. F. Callaghan, Advances in MRI-based computational neuroanatomy: from morphometry to in-vivo histology. *Curr. Opin. Neurol.* **28**, 313–322 (2015).

22. S. Genc, G. Ball, M. Chamberland, E. P. Raven, C. M. W. Tax, I. Ward, J. Y. M. Yang, M. Palombo, D. K. Jones, MRI signatures of cortical microstructure in human development align with oligodendrocyte cell-type expression. *Nat. Commun.* **16**, 3317 (2025).

23. H. Zhang, T. Schneider, C. A. Wheeler-Kingshott, D. C. Alexander, NODDI: Practical in vivo neurite orientation dispersion and density imaging of the human brain. *Neuroimage* **61**, 1000–1016 (2012).

24. E. Kirilina, S. Helbling, M. Morawski, K. Pine, K. Reimann, S. Jankuhn, J. Dinse, A. Deistung, J. R. Reichenbach, R. Trampel, S. Geyer, L. Müller, N. Jakubowski, T. Arendt, P.-L. Bazin, N. Weiskopf, Superficial white matter imaging: Contrast mechanisms and whole-brain in vivo mapping. *Sci. Adv.* **6**, eaaz9281 (2020).

25. L. Vaculčiaková, K. Podranski, L. J. Edwards, D. Ocal, T. Veale, N. C. Fox, R. Haak, P. Ehses, M. F. Callaghan, K. J. Pine, N. Weiskopf, Combining navigator and optical prospective motion correction for high-quality 500 μm resolution quantitative multi-parameter mapping at 7T. *Magn. Reson. Med.* **88**, 787–801 (2022).

26. N. Weiskopf, J. Suckling, G. Williams, M. M. Correia, B. Inkster, R. Tait, C. Ooi, E. T. Bullmore, A. Lutti, Quantitative multi-parameter mapping of R1, PD*, MT, and R2* at 3T: A multi-center validation. *Front. Neurosci.* **7**, 95 (2013).

27. A. L. Alexander, J. E. Lee, M. Lazar, A. S. Field, Diffusion tensor imaging of the brain. *Neurotherapeutics* **4**, 316–329 (2007).

28. E. Martinez-Heras, F. Grussu, F. Prados, E. Solana, S. Llufriu, Diffusion-weighted imaging: Recent advances and applications. *Semin. Ultrasound CT MR* **42**, 490–506 (2021).

29. V. S. Natu, M. Rosenke, H. Wu, F. R. Querdasi, H. Kular, N. Lopez-Alvarez, M. Grotheer, S. Berman, A. A. Mezer, K. Grill-Spector, Infants' cortex undergoes microstructural growth coupled with myelination during development. *Commun. Biol.* **4**, 1191 (2021).

30. S.-K. Song, S.-W. Sun, M. J. Ramsbottom, C. Chang, J. Russell, A. H. Cross, Dysmyelination revealed through MRI as increased radial (but Unchanged Axial) diffusion of water. *Neuroimage* **17**, 1429–1436 (2002).

31. S.-K. Song, J. Yoshino, T. Q. Le, S.-J. Lin, S.-W. Sun, A. H. Cross, R. C. Armstrong, Demyelination increases radial diffusivity in corpus callosum of mouse brain. *Neuroimage* **26**, 132–140 (2005).

32. C. Reveley, F. Q. Ye, R. B. Mars, D. Matrov, Y. Chudasama, D. A. Leopold, Diffusion MRI anisotropy in the cerebral cortex is determined by unmyelinated tissue features. *Nat. Commun.* **13**, 6702 (2022).

33. H. Fukutomi, M. F. Glasser, H. Zhang, J. A. Autio, T. S. Coalson, T. Okada, K. Togashi, D. C. Van Essen, T. Hayashi, Neurite imaging reveals microstructural variations in human cerebral cortical gray matter. *Neuroimage* **182**, 488–499 (2018).

34. C. Reveley, F. Q. Ye, D. A. Leopold, Diffusion kurtosis imaging, MAP-MRI and NODDI selectively track gray matter myelin density in the primate cerebral cortex. *Imaging Neurosci.* **2**, imag–2–00368 (2024).

35. W. Qin, Y. Liu, T. Jiang, C. Yu, The development of visual areas depends differently on visual experience. *PLOS ONE* **8**, e53784 (2013).

36. M. F. Callaghan, O. Josephs, M. Herbst, M. Zaitsev, N. Todd, N. Weiskopf, An evaluation of prospective motion correction (PMC) for high resolution quantitative MRI. *Front. Neurosci.* **9**, 97 (2015).

37. M. Zaitsev, J. Maclaren, M. Herbst, Motion artifacts in MRI: A complex problem with many partial solutions. *J. Magn. Reson. Imaging* **42**, 887–901 (2015).

38. N. C. Benson, O. H. Butt, D. H. Brainard, G. K. Aguirre, Correction of distortion in flattened representations of the cortical surface allows prediction of V1-V3 functional organization from anatomy. *PLOS Comput. Biol.* **10**, e1003538 (2014).

39. S. Yuan, M. Liu, S. Kim, J. Yang, A. J. Barkovich, D. Xu, H. Kim, Cyto/myeloarchitecture of cortical gray matter and superficial white matter in early neurodevelopment: multimodal MRI study in preterm neonates. *Cereb. Cortex* **33**, 357–373 (2022).

40. M. Wu, L. H. Lu, A. Lowes, S. Yang, A. M. Passarotti, X. J. Zhou, M. N. Pavuluri, Development of superficial white matter and its structural interplay with cortical gray matter in children and adolescents. *Hum. Brain Mapp.* **35**, 2806–2816 (2014).

41. I. Kostović, N. Jovanov-Milošević, M. Radoš, G. Sedmak, V. Benjak, M. Kostović-Srzentić, L. Vasung, M. Čuljat, M. Radoš, P. Hüppi, M. Judaš, Perinatal and early postnatal reorganization of the subplate and related cellular compartments in the human cerebral wall as revealed by histological and MRI approaches. *Brain Struct. Funct.* **219**, 231–253 (2014).

42. C. Cho, M. Chamberland, F. Rheault, D. Moyer, B. A. Landman, K. G. Schilling, Microstructural characterization of short association fibers related to long-range white matter tracts in normative development. *Hum. Brain Mapp.* **46**, e70255 (2025).

43. M. F. Glasser, T. S. Coalson, E. C. Robinson, C. D. Hacker, J. Harwell, E. Yacoub, K. Ugurbil, J. Andersson, C. F. Beckmann, M. Jenkinson, S. M. Smith, D. C. Van Essen, A multi-modal parcellation of human cerebral cortex. *Nature* **536**, 171–178 (2016).

44. M. Ouyang, J. Dubois, Q. Yu, P. Mukherjee, H. Huang, Delineation of early brain development from fetuses to infants with diffusion MRI and beyond. *Neuroimage* **185**, 836–850 (2019).

45. M. Georgiadis, A. Schroeter, Z. Gao, M. Guizar-Sicairos, M. Liebi, C. Leuze, J. A. McNab, A. Balolia, J. Veraart, B. Ades-Aron, S. Kim, T. Shepherd, C. H. Lee, P. Walczak, S. Chodankar, P. DiGiacomo, G. David, M. Augath, V. Zerbi, S. Sommer, I. Rajkovic, T. Weiss, O. Bunk, L. Yang, J. Zhang, D. S. Novikov, M. Zeineh, E. Fieremans, M. Rudin, Nanostructure-specific X-ray tomography reveals myelin levels, integrity and axon orientations in mouse and human nervous tissue. *Nat. Commun.* **12**, 2941 (2021).

46. A. Lazari, I. Lipp, Can MRI measure myelin? Systematic review, qualitative assessment, and meta-analysis of studies validating microstructural imaging with myelin histology. *Neuroimage* **230**, 117744 (2021).

47. M. Mancini, A. Karakuzu, J. Cohen-Adad, M. Cercignani, T. E. Nichols, N. Stikov, An interactive meta-analysis of MRI biomarkers of myelin. *eLife* **9**, e61523 (2020).

48. K. Schmierer, D. J. Tozer, F. Scaravilli, D. R. Altmann, G. J. Barker, P. S. Tofts, D. H. Miller, Quantitative magnetization transfer imaging in postmortem multiple sclerosis brain. *J. Magn. Reson. Imaging* **26**, 41–51 (2007).

49. S. N. Jespersen, C. R. Bjarkam, J. R. Nyengaard, M. M. Chakravarty, B. Hansen, T. Vosegaard, L. Østergaard, D. Yablonskiy, N. C. Nielsen, P. Vestergaard-Poulsen, Neurite density from magnetic resonance diffusion measurements at ultrahigh field: Comparison with light microscopy and electron microscopy. *Neuroimage* **49**, 205–216 (2010).

50. N. Wang, J. Zhang, G. Cofer, Y. Qi, R. J. Anderson, L. E. White, G. A. Johnson, Neurite orientation dispersion and density imaging of mouse brain microstructure. *Brain Struct. Funct.* **224**, 1797–1813 (2019).

51. L. Gyllensten, T. Malmfors, Myelinization of the optic nerve and its dependence on visual function— A quantitative investigation in mice. *Development* **11**, 255–266 (1963).

52. H. Tauber, T. V. Waehneldt, V. Neuhoff, Myelination in rabbit optic nerves is accelerated by artificial eye opening. *Neurosci. Lett.* **16**, 235–238 (1980).

53. Q. Li, M. Song, J. Xu, W. Qin, C. Yu, T. Jiang, Cortical thickness development of human primary visual cortex related to the age of blindness onset. *Brain Imaging Behav.* **11**, 1029–1036 (2017).

54. S. C. L. Deoni, D. C. Dean, J. Remer, H. Dirks, J. O'Muircheartaigh, Cortical maturation and myelination in healthy toddlers and young children. *Neuroimage* **115**, 147–161 (2015).

55. R. Trampel, D. V. M. Ott, R. Turner, Do the congenitally blind have a stria of gennari? first intracortical insights in vivo. *Cereb. Cortex* **21**, 2075–2081 (2011).

56. M. Fukunaga, T.-Q. Li, P. van Gelderen, J. A. de Zwart, K. Shmueli, B. Yao, J. Lee, D. Maric, M. A. Aronova, G. Zhang, R. D. Leapman, J. F. Schenck, H. Merkle, J. H. Duyn, Layer-specific variation of iron content in cerebral cortex as a source of MRI contrast. *Proc. Natl. Acad. Sci. U.S.A.* **107**, 3834–3839 (2010).

57. B. A. Barres, M. C. Raff, Proliferation of oligodendrocyte precursor cells depends on electrical activity in axons. *Nature* **361**, 258–260 (1993).

58. E. M. Gibson, D. Purger, C. W. Mount, A. K. Goldstein, G. L. Lin, L. S. Wood, I. Inema, S. E. Miller, G. Bieri, J. B. Zuchero, B. A. Barres, P. J. Woo, H. Vogel, M. Monje, Neuronal activity promotes oligodendrogenesis and adaptive myelination in the mammalian brain. *Science* **344**, 1252304 (2014).

59. N. J. Priebe, A. W. McGee, Mouse vision as a gateway for understanding how experience shapes neural circuits. *Front. Neural Circuits* **8**, 123 (2014).

60. A. W. McGee, Y. Yang, Q. S. Fischer, N. W. Daw, S. M. Strittmatter, Experience-driven plasticity of visual cortex limited by myelin and nogo receptor. *Science* **309**, 2222–2226 (2005).

61. W. Xin, M. Kaneko, R. H. Roth, A. Zhang, S. Nocera, J. B. Ding, M. P. Stryker, J. R. Chan, Oligodendrocytes and myelin limit neuronal plasticity in visual cortex. *Nature* **633**, 856–863 (2024).

62. J. S. Shimony, H. Burton, A. A. Epstein, D. G. McLaren, S. W. Sun, A. Z. Snyder, Diffusion tensor imaging reveals white matter reorganization in early blind humans. *Cereb. Cortex* **16**, 1653–1661 (2006).

63. H. Wake, F. C. Ortiz, D. H. Woo, P. R. Lee, M. C. Angulo, R. D. Fields, Nonsynaptic junctions on myelinating glia promote preferential myelination of electrically active axons. *Nat. Commun.* **6**, 7844 (2015).

64. J.-P. Bourgeois, P. Rakic, Synaptogenesis in the occipital cortex of macaque monkey devoid of retinal input from early embryonic stages. *Eur. J. Neurosci.* **8**, 942–950 (1996).

65. G. S. L. Coullon, U. E. Emir, I. Fine, K. E. Watkins, H. Bridge, Neurochemical changes in the pericalcarine cortex in congenital blindness attributable to bilateral anophthalmia. *J. Neurophysiol.* **114**, 1725–1733 (2015).

66. I. Anurova, L. A. Renier, A. G. De Volder, S. Carlson, J. P. Rauschecker, Relationship between cortical thickness and functional activation in the early blind. *Cereb. Cortex* **25**, 2035–2048 (2015).

67. M. Czarnecka, K. Hryniewiecka, A. Wolna, C. Baumbach, J. Beck, J. Vadlamudi, O. Collignon, K. Jednoróg, M. Szwed, A.-L. Stroh, Association between cortical thickness and functional response to linguistic processing in the occipital cortex of early blind individuals. *Cereb. Cortex* **35**, bhaf317 (2025).

68. A. Lutti, J. Stadler, O. Josephs, C. Windischberger, O. Speck, J. Bernarding, C. Hutton, N. Weiskopf, Robust and fast whole brain mapping of the RF transmit field B1 at 7T. *PLOS ONE* **7**, e32379 (2012).

69. F. A. Breuer, M. Blaimer, R. M. Heidemann, M. F. Mueller, M. A. Griswold, P. M. Jakob, Controlled aliasing in parallel imaging results in higher acceleration (CAIPIRINHA) for multi-slice imaging. *Magn. Reson. Med.* **53**, 684–691 (2005).

70. V. L. Yarnykh, Actual flip-angle imaging in the pulsed steady state: A method for rapid three-dimensional mapping of the transmitted radiofrequency field. *Magn. Reson. Med.* **57**, 192–200 (2007).

71. A. Lutti, C. Hutton, J. Finsterbusch, G. Helms, N. Weiskopf, Optimization and validation of methods for mapping of the radiofrequency transmit field at 3T. *Magn. Reson. Med.* **64**, 229–238 (2010).

72. D. Papp, M. F. Callaghan, H. Meyer, C. Buckley, N. Weiskopf, Correction of inter-scan motion artifacts in quantitative R1 mapping by accounting for receive coil sensitivity effects. *Magn. Reson. Med.* **76**, 1478–1485 (2016).

73. K. Tabelow, E. Balteau, J. Ashburner, M. F. Callaghan, B. Draganski, G. Helms, F. Kherif, T. Leutritz, A. Lutti, C. Phillips, E. Reimer, L. Ruthotto, M. Seif, N. Weiskopf, G. Ziegler, S. Mohammadi, hMRI – A toolbox for quantitative MRI in neuroscience and clinical research. *Neuroimage* **194**, 191–210 (2019).

74. L. J. Edwards, S. Mohammadi, K. J. Pine, M. F. Callaghan, N. Weiskopf, Robust and efficient R2* estimation in human brain using log-linear weighted least squares. *Proc. Int. Soc. Magn. Reson. Med.* **31**, 10.58530/2022/1264 (2022).

75. G. Helms, H. Dathe, P. Dechent, Quantitative FLASH MRI at 3T using a rational approximation of the Ernst equation. *Magn. Reson. Med.* **59**, 667–672 (2008).

76. L. J. Edwards, K. J. Pine, I. Lipp, E. B. C. Consortium, E. Kirilina, G. Helms, N. Weiskopf, Analytical dual flip angle R1 calculation outside the small-angle regime. *Magn. Reson. Med.* **95**, 2420–2428 (2026).

77. J. Ashburner, K. J. Friston, Unified segmentation. *Neuroimage* **26**, 839–851 (2005).

78. P. S. Tofts, "PD: proton density of tissue water" in *Quantitative MRI of the Brain: Principles of Physical Measurement* (Boca Raton: CRC Press, 2018), pp. 55–71.

79. G. Helms, H. Dathe, K. Kallenberg, P. Dechent, High-resolution maps of magnetization transfer with inherent correction for RF inhomogeneity and T1 relaxation obtained from 3D FLASH MRI. *Magn. Reson. Med.* **60**, 1396–1407 (2008).

80. M. F. Glasser, S. N. Sotiropoulos, J. A. Wilson, T. S. Coalson, B. Fischl, J. L. Andersson, J. Xu, S. Jbabdi, M. Webster, J. R. Polimeni, D. C. Van Essen, M. Jenkinson, WU-Minn HCP Consortium, The minimal preprocessing pipelines for the Human Connectome Project. *Neuroimage* **80**, 105–124 (2013).

81. J. L. R. Andersson, S. N. Sotiropoulos, An integrated approach to correction for off-resonance effects and subject movement in diffusion MR imaging. *Neuroimage* **125**, 1063–1078 (2016).

82. J. L. R. Andersson, S. Skare, J. Ashburner, How to correct susceptibility distortions in spin-echo echo-planar images: application to diffusion tensor imaging. *Neuroimage* **20**, 870–888 (2003).

83. S. M. Smith, M. Jenkinson, M. W. Woolrich, C. F. Beckmann, T. E. J. Behrens, H. Johansen-Berg, P. R. Bannister, M. De Luca, I. Drobnjak, D. E. Flitney, R. K. Niazy, J. Saunders, J. Vickers, Y. Zhang, N. De Stefano, J. M. Brady, P. M. Matthews, Advances in functional and structural MR image analysis and implementation as FSL. *Neuroimage* **23**, S208–S219 (2004).

84. K. Setsompop, B. A. Gagoski, J. R. Polimeni, T. Witzel, V. J. Wedeen, L. L. Wald, Blipped-controlled aliasing in parallel imaging for simultaneous multislice echo planar imaging with reduced g-factor penalty. *Magn. Reson. Med.* **67**, 1210–1224 (2012).

85. J.-D. Tournier, R. Smith, D. Raffelt, R. Tabbara, T. Dhollander, M. Pietsch, D. Christiaens, B. Jeurissen, C.-H. Yeh, A. Connelly, *MRtrix3*: A fast, flexible and open software framework for medical image processing and visualisation. *Neuroimage* **202**, 116137 (2019).

86. M. Jenkinson, C. F. Beckmann, T. E. J. Behrens, M. W. Woolrich, S. M. Smith, *FSL*, *NeuroImage* **62**, 782–790 (2012).

87. M. W. Woolrich, S. Jbabdi, B. Patenaude, M. Chappell, S. Makni, T. Behrens, C. Beckmann, M. Jenkinson, S. M. Smith, Bayesian analysis of neuroimaging data in FSL. *Neuroimage* **45**, S173–S186 (2009).

88. B. B. Avants, N. J. Tustison, G. Song, P. A. Cook, A. Klein, J. C. Gee, A reproducible evaluation of ANTs similarity metric performance in brain image registration. *Neuroimage* **54**, 2033–2044 (2011).

89. J. Veraart, E. Fieremans, D. S. Novikov, Diffusion MRI noise mapping using random matrix theory. *Magn. Reson. Med.* **76**, 1582–1593 (2016).

90. J. Veraart, D. S. Novikov, D. Christiaens, B. Ades-Aron, J. Sijbers, E. Fieremans, Denoising of diffusion MRI using random matrix theory. *Neuroimage* **142**, 394–406 (2016).

91. D. Perrone, J. Aelterman, A. Pižurica, B. Jeurissen, W. Philips, A. Leemans, The effect of Gibbs ringing artifacts on measures derived from diffusion MRI. *Neuroimage* **120**, 441–455 (2015).

92. J. Veraart, E. Fieremans, I. O. Jelescu, F. Knoll, D. S. Novikov, Gibbs ringing in diffusion MRI. *Magn. Reson. Med.* **76**, 301–314 (2016).

93. J. L. R. Andersson, M. S. Graham, E. Zsoldos, S. N. Sotiropoulos, Incorporating outlier detection and replacement into a non-parametric framework for movement and distortion correction of diffusion MR images. *Neuroimage* **141**, 556–572 (2016).

94. N. J. Tustison, B. B. Avants, P. A. Cook, Y. Zheng, A. Egan, P. A. Yushkevich, J. C. Gee, N4ITK: Improved N3 bias correction. *IEEE Trans. Med. Imaging* **29**, 1310–1320 (2010).

95. M. Bastiani, M. Cottaar, S. P. Fitzgibbon, S. Suri, F. Alfaro-Almagro, S. N. Sotiropoulos, S. Jbabdi, J. L. R. Andersson, Automated quality control for within and between studies diffusion MRI data using a non-parametric framework for movement and distortion correction. *Neuroimage* **184**, 801–812 (2019).

96. B. Jeurissen, J.-D. Tournier, T. Dhollander, A. Connelly, J. Sijbers, Multi-tissue constrained spherical deconvolution for improved analysis of multi-shell diffusion MRI data. *Neuroimage* **103**, 411–426 (2014).

97. J.-D. Tournier, F. Calamante, D. G. Gadian, A. Connelly, Direct estimation of the fiber orientation density function from diffusion-weighted MRI data using spherical deconvolution. *Neuroimage* **23**, 1176–1185 (2004).

98. J.-D. Tournier, F. Calamante, A. Connelly, Robust determination of the fibre orientation distribution in diffusion MRI: Non-negativity constrained super-resolved spherical deconvolution. *Neuroimage* **35**, 1459–1472 (2007).

99. R. E. Smith, J.-D. Tournier, F. Calamante, A. Connelly, SIFT: Spherical-deconvolution informed filtering of tractograms. *Neuroimage* **67**, 298–312 (2013).

100. T. Dhollander, D. Raffelt, A. Connelly, "Unsupervised 3-tissue response function estimation from single-shell or multi-shell diffusion MR data without a co-registered T1 image" in *ISMRM Workshop on Breaking the Barriers of Diffusion MRI* (Lisbon, Portugal, 2016), p. 5.

101. B. Jeurissen, A. Leemans, J.-D. Tournier, D. K. Jones, J. Sijbers, Investigating the prevalence of complex fiber configurations in white matter tissue with diffusion magnetic resonance imaging. *Hum. Brain Mapp.* **34**, 2747–2766 (2013).

102. A. L. Alexander, K. M. Hasan, M. Lazar, J. S. Tsuruda, D. L. Parker, Analysis of partial volume effects in diffusion-tensor MRI. *Magn. Reson. Med.* **45**, 770–780 (2001).

103. C. S. Parker, T. Veale, M. Bocchetta, C. F. Slattery, I. B. Malone, D. L. Thomas, J. M. Schott, D. M. Cash, H. Zhang, Not all voxels are created equal: Reducing estimation bias in regional NODDI metrics using tissue-weighted means. *Neuroimage* **245**, 118749 (2021).

104. J. P. Mugler III, J. R. Brookeman, Rapid three-dimensional T1-weighted MR imaging with the MP-RAGE sequence. *J. Magn. Reson. Imaging* **1**, 561–567 (1991).

105. A. M. Dale, B. Fischl, M. I. Sereno, Cortical surface-based analysis: I. Segmentation and surface reconstruction. *NeuroImage* **9**, 179–194 (1999).

106. B. Fischl, A. M. Dale, Measuring the thickness of the human cerebral cortex from magnetic resonance images. *Proc. Natl. Acad. Sci. U.S.A.* **97**, 11050–11055 (2000).

107. M. D. Waehnert, J. Dinse, M. Weiss, M. N. Streicher, P. Waehnert, S. Geyer, R. Turner, P.-L. Bazin, Anatomically motivated modeling of cortical laminae. *Neuroimage* **93**, 210–220 (2014).

108. K. Wagstyl, C. Paquola, R. Bethlehem, A. C. Evans, A. Huth, Equivolumetric layering for mesh surfaces [dataset]. Zenodo (2018); https://doi.org/10.5281/zenodo.1442584.

109. I. Alvarez, A. J. Parker, H. Bridge, Normative cerebral cortical thickness for human visual areas. *Neuroimage* **201**, 116057 (2019).

110. R. Huber, How many layers should I extract? *layer fMRI blog* (2019); https://layerfmri.com/2019/02/22/how-many-layers-should-i-reconstruct/.

111. M. Jenkinson, P. Bannister, M. Brady, S. Smith, Improved optimization for the robust and accurate linear registration and motion correction of brain images. *Neuroimage* **17**, 825–841 (2002).

112. B. B. Avants, C. L. Epstein, M. Grossman, J. C. Gee, Symmetric diffeomorphic image registration with cross-correlation: Evaluating automated labeling of elderly and neurodegenerative brain. *Med. Image Anal.* **12**, 26–41 (2008).

113. R Core Team, R: A Language and Environment for Statistical Computing, R Foundation for Statistical Computing (2022); https://www.R-project.org/.

114. R. V. Lenth, _emmeans: Estimated Marginal Means, aka Least-Squares Means_, version 1.8.7 (2023); https://CRAN.R-project.org/package=emmeans.

115. S. Holm, A simple sequentially rejective multiple test procedure. *Scand. J. Stat.* **6**, 65–70 (1979).

116. D. Carey, F. Caprini, M. Allen, A. Lutti, N. Weiskopf, G. Rees, M. F. Callaghan, F. Dick, Quantitative MRI provides markers of intra-, inter-regional, and age-related differences in young adult cortical microstructure. *Neuroimage* **182**, 429–440 (2018).

117. C. Stüber, M. Morawski, A. Schäfer, C. Labadie, M. Wähnert, C. Leuze, M. Streicher, N. Barapatre, K. Reimann, S. Geyer, D. Spemann, R. Turner, Myelin and iron concentration in the human brain: A quantitative study of MRI contrast. *Neuroimage* **93**, 95–106 (2014).

118. W. D. Rooney, G. Johnson, X. Li, E. R. Cohen, S.-G. Kim, K. Ugurbil, C. S. Springer Jr., Magnetic field and tissue dependencies of human brain longitudinal $^{1}H_2O$ relaxation in vivo. *Magn. Reson. Med.* **57**, 308–318 (2007).

119. B. Bender, U. Klose, The in vivo influence of white matter fiber orientation towards B_0 on T2* in the human brain. *NMR Biomed.* **23**, 1071–1076 (2010).

120. M. Cercignani, N. G. Dowell, P. S. Tofts, Eds., *Quantitative MRI of the Brain: Principles of Physical Measurement, Second Edition* (CRC Press, Boca Raton, ed. 2, 2018).

121. A. Mezer, J. D. Yeatman, N. Stikov, K. N. Kay, N.-J. Cho, R. F. Dougherty, M. L. Perry, J. Parvizi, L. H. Hua, K. Butts-Pauly, B. A. Wandell, Quantifying the local tissue volume and composition in individual brains with magnetic resonance imaging. *Nat. Med.* **19**, 1667–1672 (2013).

122. J. Annese, A. Pitiot, I. D. Dinov, A. W. Toga, A myelo-architectonic method for the structural classification of cortical areas. *Neuroimage* **21**, 15–26 (2004).

123. A. Fracasso, S. J. van Veluw, F. Visser, P. R. Luijten, W. Spliet, J. J. M. Zwanenburg, S. O. Dumoulin, N. Petridou, Lines of Baillarger in vivo and ex vivo: Myelin contrast across lamina at 7 T MRI and histology. *Neuroimage* **133**, 163–175 (2016).

124. O. Vogt, Die myeloarchitektonische Felderung des menschlichen Stirnhirns. *J. Psychol. Neurol.* **15**, 221–238 (1920).

125. F. Dick, A. Taylor Tierney, A. Lutti, O. Josephs, M. I. Sereno, N. Weiskopf, In vivo functional and myeloarchitectonic mapping of human primary auditory areas. *J. Neurosci.* **32**, 16095–16105 (2012).

126. M. I. Sereno, A. Lutti, N. Weiskopf, F. Dick, Mapping the human cortical surface by combining quantitative T1 with retinotopy. *Cereb. Cortex* **23**, 2261–2268 (2013).

127. P. Freund, N. Weiskopf, J. Ashburner, K. Wolf, R. Sutter, D. R. Altmann, K. Friston, A. Thompson, A. Curt, MRI investigation of the sensorimotor cortex and the corticospinal tract after acute spinal cord injury: A prospective longitudinal study. *Lancet Neurol.* **12**, 873–881 (2013).

128. G. Ziegler, T. U. Hauser, M. Moutoussis, E. T. Bullmore, I. M. Goodyer, P. Fonagy, P. B. Jones, U. Lindenberger, R. J. Dolan, Compulsivity and impulsivity traits linked to attenuated developmental fronto-striatal myelination trajectories. *Nat. Neurosci.* **22**, 992–999 (2019).

129. K. J. Whitaker, P. E. Vértes, R. Romero-Garcia, F. Váša, M. Moutoussis, G. Prabhu, N. Weiskopf, M. F. Callaghan, K. Wagstyl, T. Rittman, R. Tait, C. Ooi, J. Suckling, B. Inkster, P. Fonagy, R. J. Dolan, P. B. Jones, I. M. Goodyer, the NSPN Consortium, E. T. Bullmore, Adolescence is associated with genomically patterned consolidation of the hubs of the human brain connectome. *Proc. Natl. Acad. Sci. U.S.A.* **113**, 9105–9110 (2016).

130. J. D. Yeatman, B. A. Wandell, A. A. Mezer, Lifespan maturation and degeneration of human brain white matter. *Nat. Commun.* **5**, 4932 (2014).

131. M. D. Budde, M. Xie, A. H. Cross, S.-K. Song, Axial diffusivity is the primary correlate of axonal injury in the experimental autoimmune encephalomyelitis spinal cord: a quantitative pixelwise analysis. *J. Neurosci.* **29**, 2805–2813 (2009).

132. P. Rakic, J.-P. Bourgeois, M. F. Eckenhoff, N. Zecevic, P. S. Goldman-Rakic, Concurrent overproduction of synapses in diverse regions of the primate cerebral cortex. *Science* **232**, 232–235 (1986).

133. Y. Niu, M. C. Camacho, K. G. Schilling, K. L. Humphreys, In vivo mapping of infant brain microstructure with neurite orientation dispersion and density imaging. *Brain Struct. Funct.* **230**, 147 (2025).

134. B. Mota, S. Herculano-Houzel, Cortical folding scales universally with surface area and thickness, not number of neurons. *Science* **349**, 74–77 (2015).
